# Sintilimab (anti-PD-1 antibody) combined with high-dose methotrexate, temozolomide, and rituximab (anti-CD20 antibody) in primary central nervous system lymphoma: a phase 2 study

**DOI:** 10.1038/s41392-024-01941-x

**Published:** 2024-09-04

**Authors:** Zhiyong Zeng, Apeng Yang, Jingke Yang, Sheng Zhang, Zhen Xing, Xingfu Wang, Wenzhong Mei, Changzhen Jiang, Junfang Lin, Xiyue Wu, Yihui Xue, Zanyi Wu, Lianghong Yu, Dengliang Wang, Jianwu Chen, Shufa Zheng, Qiaoxian Lin, Qingjiao Chen, Jinfeng Dong, Xiaoqiang Zheng, Jizhen Wang, Jinlong Huang, Zhenying Chen, Ping Chen, Meihong Zheng, Xiaofang Zhou, Youwen He, Yuanxiang Lin, Junmin Chen

**Affiliations:** 1 Department of Hematology, the First Affiliated Hospital of Fujian Medical University, Fuzhou, China; 2 Department of Hematology, National Regional Medical Center, Binhai Campus of the First Affiliated Hospital, Fujian Medical University, Fuzhou, China; 3 Fujian Lymphoma and Multiple Myeloma Working Group, Fuzhou, China; 4 Parexel International, Durham, North Carolina USA; 5 Department of Pathology, the First Affiliated Hospital of Fujian Medical University, Fuzhou, China; 6 Department of Imaging, the First Affiliated Hospital of Fujian Medical University, Fuzhou, China; 7 Department of Neurosurgery, the First Affiliated Hospital of Fujian Medical University, Fuzhou, China; 8 Department of Nuclear Medicine, the First Affiliated Hospital of Fujian Medical University, Fuzhou, China; 9 Beijing tricision Biotherapeutics Inc., Beijing, China

**Keywords:** CNS cancer, Drug development

## Abstract

Primary central nervous system lymphoma (PCNSL) is a rare and frequently fatal lymphoma subtype. The programmed death-1 (PD-1) pathway has emerged as a potential therapeutic target, but the effectiveness of PD-1 antibody sintilimab in combination with immunochemotherapy as a frontline treatment for PCNSL remains to be determined. In this phase 2 trial (ChiCTR1900027433) with a safety run-in, we included patients aged 18–70 with newly diagnosed PCNSL. Participants underwent six 21-day cycles of a SMTR regimen, which includes sintilimab (200 mg, Day 0), rituximab (375 mg/m^2^, Day 0), methotrexate (3.0 g/m^2^, Day 1 or 1.0 g/m^2^ for patients aged ≥65 years), and temozolomide (150 mg/m^2^/d, Days 1–5). Among 27 evaluable patients, the overall response rate (ORR) was 96.3% (95% confidence interval: 81–99.9%), with 25 complete responses. At a median follow-up of 24.4 months, the medians for duration of response, progression-free survival (PFS), and overall survival were not reached. The most common grade 3–4 treatment-related toxicities were increased levels of alanine aminotransferase (17.9%) and aspartate aminotransferase (14.3%). Additionally, baseline levels of interferon-α and the IL10/IL6 ratio in cerebrospinal fluid emerged as potential predictors of PFS, achieving areas under the curve of 0.88 and 0.84, respectively, at 2 years. Whole-exome sequencing revealed a higher prevalence of RTK-RAS and PI3K pathway mutations in the durable clinical benefit group, while a greater frequency of Notch and Hippo pathway mutations in the no durable benefit group. These findings suggest the SMTR regimen is highly efficacious and tolerable for newly diagnosed PCNSL, warranting further investigation.

## Introduction

Primary central nervous system lymphoma (PCNSL), predominantly a diffuse large B-cell lymphoma (DLBCL) subtype, presents unique clinical challenges due to its aggressive nature. Historically, median overall survival (OS) for PCNSL patients was only 1.3 years.^1^ However, recent advancements in treatment have extended this to 25.3 months.^2^ High-dose methotrexate (HD-MTX)-based regimens remain the cornerstone of front-line induction therapy for PCNSL, often followed by consolidation strategies like radiation or autologous stem cell transplantation (ASCT) to prolong the response duration. In a phase 2 trial, patients undergoing intensive combination therapies, such as high-dose chemotherapy followed by ASCT (HDC-ASCT), demonstrated significantly higher survival rates, with a 5-year OS rate of approximately 79%.^3^ Despite these efforts, relapse is common and 5-year survival rates remain around 30% to 40% in real-world settings,^4^ highlighting the urgent need for innovative treatments. The optimal combination of medications with methotrexate (MTX) remains undetermined due to the paucity of head-to-head clinical trials.^4^ Recent research has focused on integrating rituximab into HD-MTX-based regimens, as evidenced by several clinical trials for PCNSL.^5–8^

In 2012, Wieduwilt and colleagues pioneered the MTR regimen (MTX (8.0 g/m^2^), temozolomide, rituximab), followed by high-dose consolidation using etoposide and cytarabine (EA).^9^ This approach achieved an overall response rate (ORR) of 58% and a complete response (CR) rate of 52%, demonstrating initial success in PCNSL management. Subsequent studies, including the CALGB 50202 trial and retrospective analyses,^5,10^ validated these findings but also revealed significant challenges, notably a high discontinuation rate of 48-54% due to progression and considerable treatment-related toxicities.^5,9,10^ Grade 4 neutropenia and thrombocytopenia were prevalent, affecting 81–100% of patients undergoing EA consolidation. These findings highlight the need to refine the MTR + EA regimen to improve tolerability and patient adherence, which is a key focus of our current research.

Targeting the programmed cell death-1 (PD-1)/PD-ligand 1 (PD-L1) pathway in combination with rituximab has exhibited synergistic anti-cancer effect. Two phase 2 studies of PD-1 blockade by pidilizumab or pembrolizumab in combination with rituximab, an anti-CD20 antibody, in patients with relapsed or refractory follicular lymphoma demonstrated an objective response of 67%.^11,12^ Temozolomide has been observed to induce PD-L1 expression in tumor cells, facilitating immune evasion.^13^ The combined administration of temozolomide and a PD-1 antibody significantly reduced tumor size and enhanced the infiltration of CD4 and CD8 cells into brain tumors.^14^ Notably, frequent increases in the copy numbers and elevated expression of 9p24.1/PD-L1 and PD-L2 in PCNSL are now recognized as independent prognostic factors for poor patient outcomes,^15,16^ highlighting the critical role of the PD-1/PD-L1 pathway in PCNSL. Encouraging therapeutic efficacy of PD-1 antibody on central nervous system (CNS) lymphoma has been observed in preclinical study and case series report of salvage treatments.^17–19^ Gavrilenko et al.’s study further clarifies the effectiveness of PD-1 antibody-based treatment for primary large B-cell lymphoma of immune-privileged sites (PLBLIPS) and secondary CNS lymphoma (SCNSL), showing promising ORRs of 71% and 67%, respectively.^20^ Taken together, these findings support the potential synergistic effect of PD-1-blocking treatment in combination with MTR induction regimen for PCNSL management.

Sintilimab, a humanized IgG4 monoclonal antibody specific for human PD-1, was firstly approved by the National Medical Products Administration in China for treating relapsed or refractory Hodgkin lymphoma after at least two lines of systemic chemotherapy.^21^ Here, we present the efficacy and safety of sintilimab combined with HD-MTX, temozolomide, and rituximab (SMTR) from a single-arm phase 2 trial in patients with PCNSL as a first-line treatment.

## Results

### Characteristics of patients

**Fig. 1:**
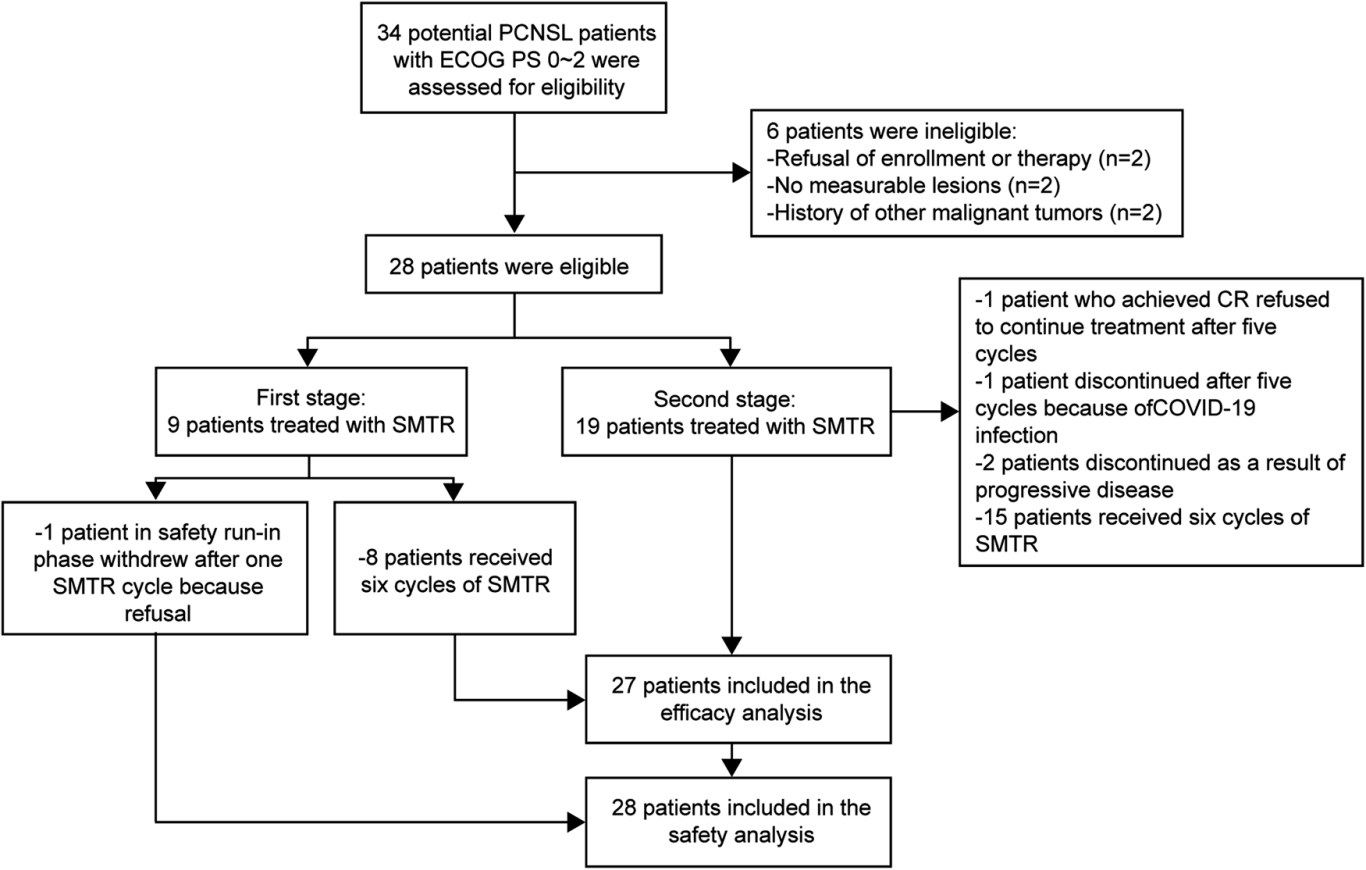
Trial profile. CR, complete response; ECOG PS, Eastern Cooperative Oncology Group performance status; SMTR, sintilimab combined with high-dose methotrexate, temozolomide and rituximab; PCNSL, primary central nervous system lymphoma; SCNSL, secondary central nervous system lymphoma

From 3 April 2020 and 7 August 2022, we evaluated 34 consecutively newly diagnosed PCNSL patients with Eastern Cooperative Oncology Group (ECOG) performance status (PS) of 0–2 for eligibility. Ultimately, 28 patients were recruited (Fig. 1). During the safety run-in phase, one patient withdrew due to refusal to continue treatment before dose-limiting toxicity (DLT) and response assessment after completing one cycle of treatment, and was subsequently replaced. The baseline characteristics of the 27 evaluable patients are summarized in Table 1. The median age was 54 years (range: 18–68 years), with 63% female. Among the 27 patients, 13 (48.1%) experienced cerebral herniation, 2 (7.4%) exhibited positive cerebrospinal fluid (CSF) cytology suggesting meningeal involvement, and 3 (11.1%) presented with intraocular disease. Additionally, 10 (37.0%) patients displayed an ECOG PS of 2, and 20 (74.1%) had International Extranodal Lymphoma Study Group (IELSG) risk scores of ≥2.

**Table 1:** Characteristics of Patients at Baseline

Characteristic	Patients (n = 27)
Age at enrollment	
Median (range) – yr	54 (18-68)
<65 yr – no.(%)	25 (92.6%)
Sex	
Female	17 (63.0%)
Male	10 (37.0%)
Increased serum LDH	3 (11.1%)
Increased protein in CSF*	14 (51.9%)
CNS sites of disease	
Brain parenchyma only	22 (81.5%)
Brain and meninges	2 (7.4%)
Brain and intraocular disease^†^	3 (11.1%)
Positive CSF cytology	2 (7.4%)
Multiple lesions	14 (51.9%)
Deep-brain lesions	17 (63.0%)
Cerebral herniation	13 (48.1%)
ECOG performance status	
0	9 (33.3%)
1	8 (29.7%)
2	10 (37.0%)
IELSG risk group	
Low (0–1)	7 (25.9%)
Intermediate (2–3)	16 (59.3%)
High (4–5)	4 (14.8%)
Histology	
GCB	9 (33.3%)
Non-GCB	18 (66.7%)
PD-L1 expression	
>10%	14 (51.9%)
≤10%	10 (37.0%)
Not known	3 (11.1%)

^*^Data regarding the protein concentration in CSF were available for 24 patients

^†^All patients with intraocular lymphoma had concomitant brain lesions

*CNS* central nervous system, *CSF* cerebrospinal fluid, *ECOG* Eastern Cooperative Oncology Group, *GCB* germinal center B-cell, *IELSG* International Extranodal Lymphoma Study Group, *LDH* lactate dehydrogenase, *PD-L1* programmed death-ligand 1

### Response and survival

**Fig. 2:**
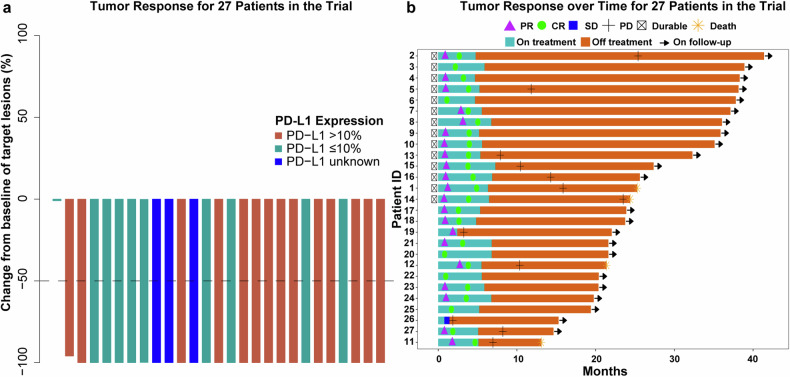
Clinical responses to a combination of sintilimab plus methotrexate, temozolomide, and rituximab in patients with newly diagnosed PCNSL. **a** The percent change from baseline of target lesions in the 27 patients who underwent radiology evaluation after treatment initiation. PD-L1 expression in tumor cells is characterized by three categories: those with PD-L1 expression levels > 10%, those with PD-L1 expression levels ≤10%, and those with unknown PD-L1 expression levels. Dashed line indicates response criteria of the IPCG for partial response (–50%). **b** Swimmer plot of responses to and duration of treatment in our study. Each bar represents one patient. CR, complete response; IPCG, International Primary Central Nervous System Lymphoma Collaborative Group; PCNSL, primary central nervous system lymphoma; PD-L1, programmed death-ligand 1; PD, progressive disease; PR, partial response; SD, stable disease

Response to the SMTR regimen is depicted in Fig. 2. Among the 27 patients who underwent at least one post-baseline tumor assessment, we observed CR in 25 (92.6%), partial response (PR) in one patient (3.7%), and stable disease (SD) in another patient (3.7%) at the end of the treatment. This resulted in an ORR of 96.3% [95% confidence interval (CI): 81–99.9%] (Fig. 2). Two patients (8.3%) aged ≥65 years with normal renal function achieved CR. Of these, 23 (85.2%) patients successfully completed all six cycles of therapy. One patient, aged >65 years, who achieved a CR voluntarily chose to discontinue treatment after five cycles. Another patient discontinued after five cycles due to COVID-19 infection, and two patients discontinued due to disease progression.

**Fig. 3:**
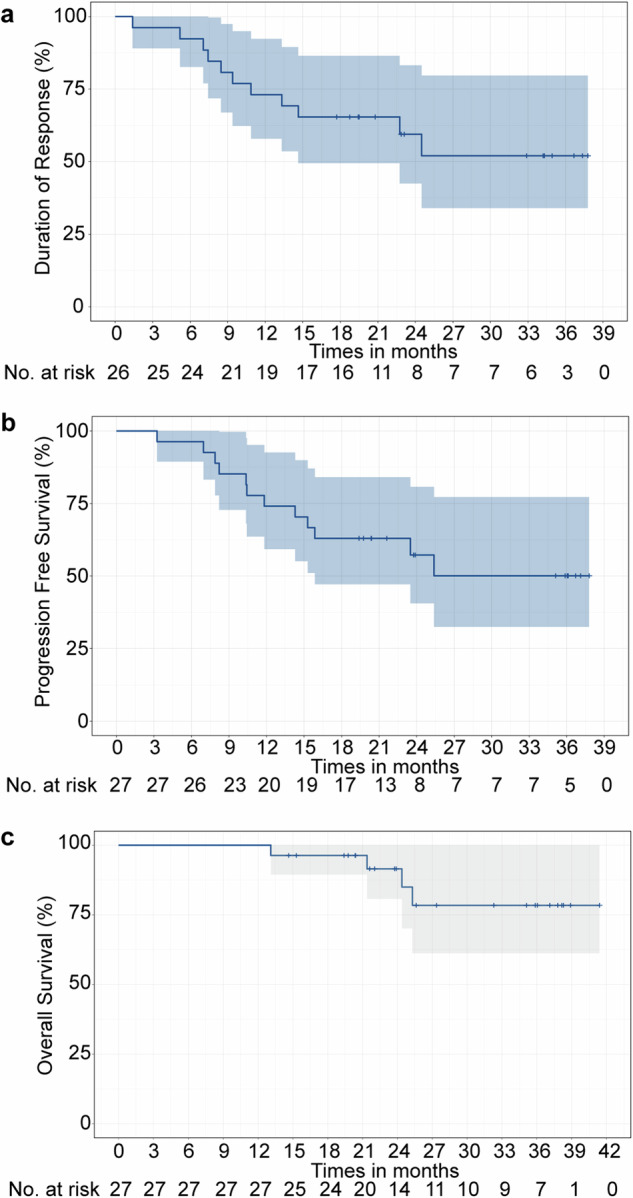
Kaplan–Meier plots of duration of response **a**, progression-free survival **b**, and overall survival **c**

As of the data cut-off on 7 October 2023, the median follow-up duration was 24.4 months (range: 13.1–41.2 months). Four patients died due to disease progression. The medians for duration of response (DOR), progression-free survival (PFS), and OS were not reached (Fig. 3). Additionally, the PFS and OS rates at the two-year mark were 57.2% (40.6–80.8%) and 91.5% (80.7–100%), respectively.

### Safety

Adverse events (AEs) are detailed in Table 2. While all 28 patients experienced treatment-related adverse events (TRAEs), it is important to note that certain events such as headaches in four patients were assessed as related to the underlying PCNSL rather than the treatment itself. Dose-limiting toxicities (DLTs) were not observed in the safety run-in cohort. The most common TRAEs across all grades were increased levels of alanine transaminase (ALT) (78.6%) and aspartate transaminase (AST) (in 75.0% of patients), leukopenia (67.9%), fatigue (53.6%), and anorexia (50.0%).

**Table 2:** Adverse Events (*n* = 28)

Events, number. (%)	Any grade	Grade 1–2	Grade 3	Grade 4	TRAEs
**Hematological toxicities**					
Leukopenia	19 (67.9%)	17 (60.7%)	0 (0%)	2 (7.2%)	Yes
Neutropenia	13 (46.4%)	11 (39.3%)	0 (0%)	2 (7.2%)	Yes
Anemia	5 (17.9%)	3 (10.7%)	1 (3.6%)	1 (3.6%)	Yes
Thrombocytopenia	7 (25.0%)	5 (17.8%)	1 (3.6%)	1 (3.6%)	Yes
Febrile neutropenia	2 (7.2%)	0 (0%)	2 (7.2%)	0 (0%)	Yes
**Non-hematological toxicities**					
Headache	4 (14.3%)	4 (14.3%)	0 (0%)	0 (0%)	No
Nausea	13 (46.4%)	13 (46.4%)	0 (0%)	0 (0%)	Yes
Vomiting	12 (42.9%)	12 (42.9%)	0 (0%)	0 (0%)	Yes
Anorexia	14 (50.0%)	14 (50.0%)	0 (0%)	0 (0%)	Yes
Abdominal pain	1 (3.6%)	1 (3.6%)	0 (0%)	0 (0%)	Yes
Diarrhea	2 (7.2%)	2 (7.2%)	0 (0%)	0 (0%)	Yes
Constipation	2 (7.2%)	2 (7.2%)	0 (0%)	0 (0%)	Yes
Fatigue	15 (53.6%)	15 (53.6%)	0 (0%)	0 (0%)	Yes
Arrhythmias	1 (3.6%)	1 (3.6%)	0 (0%)	0 (0%)	Yes
Fever	3 (10.7%)	3 (10.7%)	0 (0%)	0 (0%)	Yes
Infection in the upper respiratory tract	2 (7.2%)	2 (7.2%)	0 (0%)	0 (0%)	Yes
Pulmonary infection	3 (10.7%)	0 (0%)	3 (10.7%)	0 (0%)	Yes
Interstitial pneumonia	2 (7.2%)	2 (7.2%)	0 (0%)	0 (0%)	Yes
Increased bilirubin in blood	4 (14.3%)	4 (14.3%)	0 (0%)	0 (0%)	Yes
Increased alanine aminotransferase	21 (75.0%)	16 (57.1%)	5 (17.9%)	0 (0%)	Yes
Increased aspartate aminotransferase	22 (78.6%)	18 (64.3%)	4 (14.3%)	0 (0%)	Yes
Increased creatinine in blood	10 (35.7%)	10 (35.7%)	0 (0%)	0 (0%)	Yes
Pruritus	8 (28.6%)	8 (28.6%)	0 (0%)	0 (0%)	Yes
Herpes zoster	1 (3.6%)	1 (3.6%)	0 (0%)	0 (0%)	Yes
Rash maculo-papular	2 (7.2%)	2 (7.2%)	0 (0%)	0 (0%)	Yes

TRAEs, treatment-related adverse events

Grade 3 non-hematologic TRAEs were observed in 9 (32.1%) of patients, including 5 patients with increased ALT (17.9%) and 3 patients with increased AST (14.3%). These transaminase elevations were generally reversible with liver protection therapies (such as bicyclol and/or polyene phosphatidylcholine) and did not require corticosteroid intervention. The study treatment was continued following grade 3 transaminitis, as we attributed the potential causative role to high-dose methotrexate rather than sintilimab. Grade 4 hematological toxicity related to the treatment was observed in two patients (7.4%), while no grade 4 non-hematological toxicity was recorded.

Out of the 28 patients, 10 (35.7%) experienced grade 1 or 2 immune-related AEs. Pruritus was the most common, affecting 8 patients, 2 of whom also developed grade 1–2 rash. Additionally, 2 patients experienced grade 1 interstitial pneumonia, one of whom had fever. To manage the rash, fever and interstitial pneumonia, 4 patients (14.3%) required systemic corticosteroids (methylprednisolone), mainly during the second to third cycles (Supplementary Table S1). The median initial dose of methylprednisolone was 20 mg/day (range: 8–60 mg/day), with a median treatment duration of 7 days (range: 7–21 days). The regimen involved starting at a higher dose, which was gradually tapered until discontinuation. All immune-related AEs resolved upon the completion of the SMTR regimen. No patients reduced their dose or discontinued treatment due to the toxicity of the medications.

### Correlation of biomarkers with clinical outcomes

We assessed the influence of clinical prognostic factors on the treatment outcomes of patients undergoing the SMTR regimen. Neither ECOG PS of 2 nor a high IELSG score (4–5) had significant impact on ORR and PFS (Supplementary Fig. S1a, b). Similarly, tumor cells expressing PD-L1 were not associated with PFS (Supplementary Fig. S1c).

**Fig. 4:**
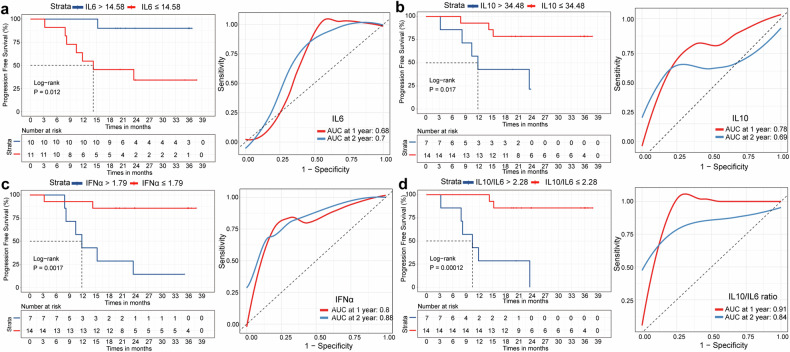
Prognostic evaluation of the concentration of IL6, IL10, IFNα, and IL10/IL6 ratio in CSF at baseline in patients with PCNSL. **a**, **b**, **c**, and **d** show Kaplan–Meier survival curves and time-dependent ROC analysis for patients based on the concentrations of IL6, IL10, IL10/IL6 ratio, and IFNα in CSF, respectively. The optimal cut-off points of cytokines as determined by ROC analysis were calculated and was defined as the threshold. AUC, area under the curve; CSF, cerebrospinal fluid; IFNα, interferon-α; IL6, interleukin 6; IL10, interleukin 10; PCNSL, primary central nervous system lymphoma; ROC, receiver operating characteristic

Subsequently, we analyzed cytokine levels in the CSF at baseline from 21 available samples. The optimal cut-off points for each cytokine as determined by ROC analysis were calculated and defined as the threshold. The baseline CSF levels of several cytokines, including (interleukin (IL)1β, IL2, IL5, IL8, IL12P70, IL17, tumor necrosis factor (TNF), and interferon-γ (IFNγ), did not show a significant correlation with PFS (Supplementary Fig. S2). However, lower levels of IL4 ( ≤ 0.83 pg/ml) and IL6 ( ≤ 14.58 pg/ml) in the CSF at baseline were associated with poorer PFS (median PFS: 14.3 months vs not reached, P < 0.05, Supplementary Fig. S2C; and median PFS: 14.3 months vs not reached, P < 0.05, Fig. 4a, respectively). Conversely, higher baseline CSF levels of IL10 ( > 34.48 pg/ml) and interferon-α (IFNα) ( > 1.79 pg/ml) were correlated with poorer PFS (median PFS: 11.8 months vs not reached, P < 0.05, Fig. 4b; median PFS: 11.8 months vs not reached, P < 0.01, Fig. 4c, respectively). Moreover, patients with a higher baseline CSF IL10/IL6 ratio ( > 2.28) were more likely to experience disease progression (median PFS: 11.8 months vs. not reached, P < 0.001, Fig. 4d). A further time-dependent receiver operating characteristic (ROC) analysis revealed that the level of IFNα had an area under the curve (AUC) of 0.88 for 2-year PFS, outperforming IL4 (AUC = 0.41), IL6 (AUC = 0.7), IL10 (AUC = 0.69), and the IL10/IL6 ratio (AUC = 0.84) (Supplementary Fig. S2d, Fig. 4).

### Genomic profiling

**Fig. 5:**
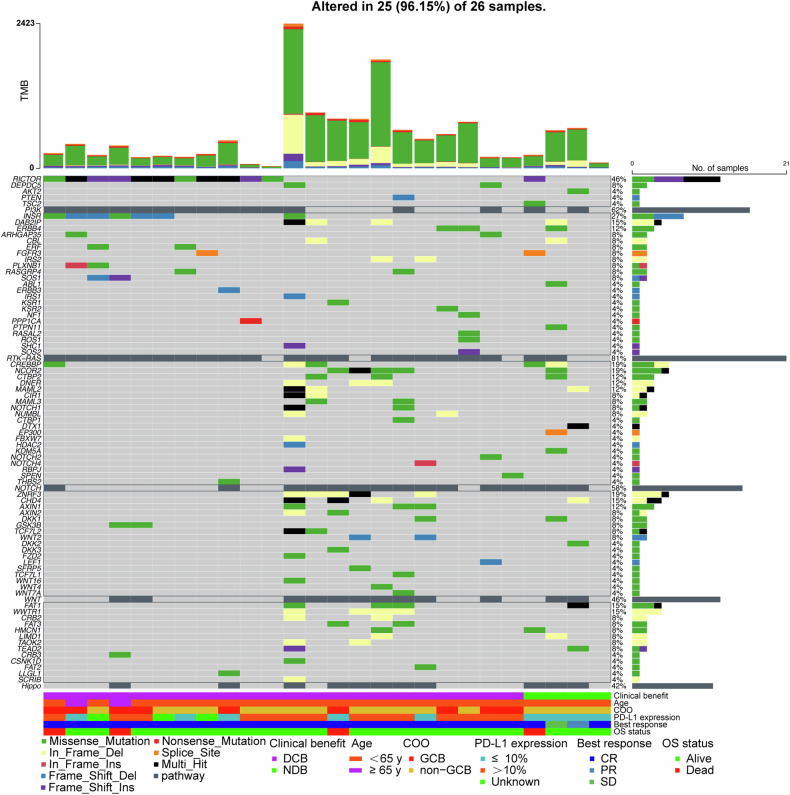
Whole-exome sequencing reveals genomic profiling and top 5 signaling pathways on FFPE tumor and matched peripheral blood samples obtained from 26 eligible patients. COO, cell of origin; CR, complete response; DCB, durable clinical benefit; FFPE, formalin-fixed paraffin-embedded; GCB, germinal Center B-cell-like; NDB, no durable benefit; Non-GCB, non-Germinal Center B-cell-like; OS, overall survival; PR, partial response; PD-L1, programmed death-ligand 1

In this study, we performed whole-exome sequencing (WES) on samples from 26 PCNSL patients to comprehensively characterize their mutational landscape, aiming to uncover genetic determinants of clinical outcomes. The analysis revealed distinct mutational patterns between patient groups, indicating potential pathways influencing treatment efficacy (Fig. 5). Specifically, mutations within the RTK-RAS and PI3K pathways were identified in 81.8% (18 out of 22 samples) and 72.7% (16 out of 22 samples) of the samples, respectively, in the durable clinical benefit (DCB) group. In contrast, these mutations occurred in 50% of samples (2 out of 4 samples) for both pathways in the no durable benefit (NDB) group. Conversely, the NDB group displayed increased frequencies of mutations in the Notch (75%, 3 out of 4 samples) and Hippo (100%, 4 out of 4 samples) pathways, compared to the DCB group, which showed lower frequencies at 54.5% (12 out of 22 samples) for Notch and 40.9% (9 out of 22 samples) for Hippo, respectively. The mutation rates of INSR, ERF, IRS2, PLXNB1, RASGRP4, SOS1 and RICTOR were higher in the DCB group compared to the NDB group. These findings suggest potential molecular mechanisms underlying the differential treatment responses observed in PCNSL patients receiving PD-1 antibody therapy.

## Discussion

To our knowledge, this phase 2 trial represents the first prospective investigation into the use of a PD-1-blocking antibody combined with immunochemotherapy MTR regimen for previously untreated PCNSL patients. The regimen demonstrated promising anti-tumor activity and an acceptable safety profile.

The higher response rate with SMTR regimen may be attributed to the immune modulation effects of one or more drugs within the MTR combination, which potentially enhance the effect of sintilimab. Previous studies have demonstrated the synergistic anti-tumor effect of anti-PD1 antibody in combination with rituximab or temozolomide.^11–14^ A retrospective analysis has identified high PD-1 expression in tumor infiltrating lymphocytes (TILs) as a significant adverse prognostic factor for PCNSL patients undergoing HD-MTX treatment.^22^ Our findings suggest that adding PD-1 antibody to the MTR regimen may counteract the adverse prognosis associated with PD-1/PD-L1 pathway.

We adjusted the MTR regimen dosage and schedule from previous protocols, reducing HD-MTX from 8.0 g/m^2^ to 3.0 g/m^2^ and altering temozolomide dosing schedule. While HD-MTX is the cornerstone in treating PCNSL, its optimal dosage remains debated. Previous studies have revealed no significant improvement in PFS or OS when using higher dosages of HD-MTX (8.0 g/m^2^) over moderate doses (3.0–3.5 g/m^2^).^23,24^ Additionally, we incorporated temozolomide on Day 1–5 of each cycle instead of Day 7–11 in the odd 14-day cycle, considering a potentially better synergistic effect of administering MTX and temozolomide on the same period, as well as shortening the treatment duration.

For elderly patients, we adjusted the MTX dose to 1.0 g/m² to balance treatment efficacy with patient safety and overall patient tolerance. This decision is supported by findings from a systemic review,^25^ which showed no significant OS advantage with HD-MTX protocols at ≥3.0 g/m^2^ compared to those with <3.0 g/m^2^. The results are consistent regardless of whether the dose cutoff value used for HD-MTX is 1.0 g/m^2^ or 3.0 g/m^2^.^25^ Furthermore, expert consensus on the management of PCNSL in China (2019) advises against routine use of HD-MTX for patients aged 60 years and older due to increased risks and reduced tolerability.^26,27^ This recommendation supports our approach to reduce the MTX dose to 1.0 g/m^2^ in older patients, highlighting the importance of tailoring chemotherapy regimens to improve patient safety and quality of life. Although only two patients aged 65 and above were included, both achieved CR with the adjusted MTX dose. This finding suggests that lower doses may still be effective in achieving significant clinical outcomes and warrants further investigation into optimal MTX dosages for elderly patients.

A pivotal adjustment in our treatment approach involves omission of the consolidation strategy, which deviates from the established international guidelines. In contrast to the established practice of administering EA consolidation after 4 cycles of MTR regimen induction,^5,9,10^ our study adopts a novel approach whereby patients achieving CR after 6 cycles of SMTR induction receive no further consolidation treatment unless disease progression occurs. This decision was based on a critical analysis of the balance between efficacy and patient safety, especially considering the significant toxicity and risk of treatment-related mortality (TRM) associated with conventional consolidation treatments. Traditional post-induction intensive chemotherapy protocols, such as those used in the CALGB 50202 trial, have been associated with a high incidence (81%) of severe hematological toxicities, including life-threatening conditions such as grade 5 sepsis.^5^ In recent years, HDC-ASCT has been a frequently considered consolidation strategy for young and fit patients.^4^ However, the incidence of early TRM within the HDC-ASCT group has been observed across several pivotal studies, including the randomized trials MATRix/IELSG43 and PRECIS, as well as in a retrospective analysis involving thiotepa-based conditioning.^28–30^ Furthermore, the recently updated 7-year results of the MATRix/IELSG32 study,^31^ reported that although patients treated with MATRix and consolidation had a 7-year OS of 70%, 6.8% of patients experienced toxic deaths during induction chemotherapy and ASCT, highlighting the necessity for meticulous patient monitoring in the administration of intensive chemotherapy. The recent study Alliance 51101 indicated similar PFS and OS between myeloablative and nonmyeloablative consolidation approaches, with a notable TRM rate of 11.4% observed in the 70 patients who completed consolidation.^32^ In addition, the MARTA study,^33^ specifically focused on older but fit patients over 65 years, reported a TRM rate of 6% associated with HDC-ASCT. In contrast, our study observed only two instances (7.4%) of grade 4 hematological toxicity in our patients, with no grade 5 events, suggesting that our modified approach could reduce toxicity significantly without compromising treatment efficacy.

Moreover, the rationale for omitting consolidation also stems from the evolving landscape of PCNSL management, aiming to improve quality of life along with extending survival. Our findings highlight the need for alternative strategies that may offer a better balance of risks and benefits. Given the preliminary nature of our results, we advocate for further clinical trials comparing different consolidation strategies to build a robust dataset guiding future treatment protocols.

As an early-phase study, our primary endpoint was the response rate, which is crucial for evaluating the preliminary efficacy of the treatment regimen. However, it is important to note that remission rates in PCNSL are assessed differently across various studies, and several reports have indicated that the depth of remission may not always correlate with OS or PFS due to factors such as minimal residual disease and patient-specific characteristics.^2,34,35^ This underscores the complexity of using remission status as the sole indicator of treatment success. While our study offers valuable insights into the potential benefits of the SMTR regimen, the results should be interpreted with caution. Future studies should aim to explore the multiple factors that influence survival to gain more complete picture of treatment outcomes in PCNSL. This approach will allow us to compare our findings with other international research, especially concerning long-term outcomes and survival.

The SMTR regimen was well tolerated in the phase 2 study, with no DLTs observed in the safety run-in cohort. Transaminitis was the most common grade 3 TRAE, whereas its prevalence was similar to that reported in other studies using the MTR regimen.^5,9,36^ Elevated transaminase levels could be managed using conventional liver-protective medications, eliminating the need for steroids. We attributed these liver toxicities to MTR regimen rather than sintilimab. Notable immune-related AEs, including pruritus, rash, and interstitial pneumonia, are controllable and reversible. Importantly, no dose reductions or treatment discontinuations occurred due to medication toxicity. Thus, the novel SMTR protocol offers potential advantages by reducing MTX dosage, avoiding intensive consolidation, ensuring efficacy, and enhancing tolerance. Although further extensive studies are needed, these findings underscore the promising aspects of our adjusted treatment strategy.

While the patients in our study were younger than those in the prospective CALGB 50202 study (median age: 54 years versus 61 years), our study had a higher proportion of patients with an ECOG PS of 2 and those in the high IELSG risk group (score 4-5). Additionally, due to the modifications made to the original MTR regimen, we cannot directly attribute the superior response rate and tolerance to the addition of sintilimab to the MTR immunotherapy. However, we hypothesize that the potential immune modulation effects of these novel immunochemotherapy combinations, along with the adjustment for the MTR regimen, at least partially contribute to these superior outcomes. Our study also showed improved tolerability compared to previous trials.^5–8^ Overall, although the synergistic effect of sintilimab with immunochemotherapy needs further validation in future randomized studies, our findings suggest anti-PD1 therapy may enhance the efficacy of MTX-based immunochemotherapy, even with a reduced treatment intensity.

Currently, there are limited reports on biomarkers predicting the prognosis of PCNSL patients treated with immune checkpoint inhibitors. While PD-L1 expression has been linked to survival and the efficacy of anti-PD-1 antibodies in certain solid tumors,^37^ our study did not reveal an apparent association in PCNSL. This could be attributed to the relatively high response rate or small sample size. Therefore, a larger patient cohort is needed to conclusively determine the discriminatory power of this test.

Our study demonstrated that elevated baseline levels of specific cytokines in CSF were associated with a poor 2-year PFS in patients with PCNSL treated with the SMTR regimen. IFNα had the greatest discriminatory power in predicting 2-year PFS, followed by the IL10/IL6 ratio. Notably, a recent study identified a critical role for IFNα in regulating immunosuppression in head and neck squamous cell carcinoma, where IFNα was shown to activate the transcription of PD-L1 via p-Stat1 (Tyr701), leading to increased expression of PD-L1.^38^ PD-L1 has also been shown to induce Type I interferon (IFN-I) synthesis in cancer cells while concurrently suppressing their ability to respond to IFN-I.^39^ Thus, our findings indicate IFNα and PD-L1 may exhibit intricate interactions in PCNSL that influence tumor progression and IFNα may serve as a potential biomarker for predicting PFS after chemoimmunotherapy. Furthermore, the high IL-10/IL-6 ratio in PCNSL patients undergoing PD-1 antibody therapy suggests an intricate interplay between these cytokines. IL-10’s anti-inflammatory properties may compromise PD-1 blockade efficacy, while IL-6’s pro-inflammatory role promotes immune responses. The dysregulation, indicated by an elevated IL-10/IL-6 ratio, may create a less conducive tumor microenvironment for PD-1 antibody therapy. Understanding IL-10 and IL-6 contributions is pivotal for refining therapeutic strategies in PCNSL.

Complementing the cytokine analysis, we employed WES to investigate signaling pathways affected by gene mutations, aiming to identify predictive biomarkers for immunotherapy with PD-1 antibody therapy. The RTK-RAS and PI3K-AKT signaling pathways, are known for their oncogenic properties in promoting PCNSL, partly through the induction of abundant cytokines and chemokines.^40,41^ We observed that alterations in the RTK/Ras and PI3K/AKT pathway components of PCNSL may render tumors more sensitive to SMTR regimen. This finding is consistent with previous studies reporting that similar alterations in these pathways in gliomas, another type of brain tumor, are associated with increased responsiveness to PD-1 antibody therapy.^41^ However, our study also suggests that the Notch and Hippo signaling pathways may serve as potential markers for predicting the lack of durable benefit from chemotherapy combined with PD-1 immunotherapy. This could be potentially linked to immune deficiency resulting from aberrations in these pathways in PCNSL.^42^

Despite the promising outcome and tolerability we observed, our study has several limitations. Primarily, as an early-phase clinical trial, the sample size is small and potentially heterogeneous, and patients were not randomized. The single-center design may also limit the generalizability of our findings. Furthermore, the absence of blinded reviews and the open-label nature of the study raise concerns about the robustness of our findings. Notably, the administration of cortisone to some patients due to AEs poses challenges to the accurate assessment of treatment response. This introduces a potential confounding factor that needs careful consideration when interpreting our results. In addition, the limited long-term follow-up data hinders our ability to fully evaluate the durability of the treatment, considering the common occurrence of late relapses in PCNSL. Lastly, future studies with larger sample sizes are warranted to further elucidate the predicting value of these CSF biomarkers and WES results.

In summary, the combination of sintilimab and the MTR regimen has potential to be highly efficacious and well tolerated in patients with newly diagnosed PCNSL, suggesting that this regimen merits further, more rigorous investigation, ideally randomized trial, to assess its potential role in the treatment of this challenging disease.

## Materials and Methods

### Patients

Patients were required to meet the following key inclusion criteria: age between 18 and 70 years, confirmed diagnosis of DLBCL via histology, exclusive localization of the disease in the brain, leptomeninges, spinal cord, CSF, and/or eyes, and an ECOG PS from 0 to 2. Other key inclusion criteria included: an estimated time of survival >12 weeks; at least one measurable lesion based on magnetic resonance imaging or positive CSF cytology; absolute neutrophil count ≥1.5 × 10^9^/L; platelet count ≥100 × 10^9^/L; levels of ALT and AST ≤ 2 × upper limit of normal (ULN); serum total bilirubin ≤1.5 × ULN; creatinine clearance ≥50 mL/min.

Key exclusion criteria include: previous immune checkpoint therapy, radiotherapy or chemotherapy for PCNSL, history of other malignancies, human immunodeficiency virus infection, active infection, and interstitial lung disease. The full list of inclusion/exclusion criteria is in the trial protocol.

### Study oversight

The protocol was approved by the Ethics Review Committee of The First Affiliated Hospital of Fujian Medical University (FAH-FMU) (Approval No.: MRCTA, ECFAH of FMU [2019]231) and conducted in accordance with the Declaration of Helsinki. The authors ensured data accuracy and adherence to the study protocol. The first author (principal investigator) and Ethics Review Committee of FAH-FMU oversaw the study. Data collection and trial procedures were managed by the trial investigators and Clinical Trials Office of FAH-FMU. Participation in the study was voluntary, and written informed consent was mandatory for all patients. The study is registered on www.chictr.org.cn (Chinese Clinical Trial Registry number: ChiCTR1900027433).

### Study design

This was a single-center, investigator-sponsored, phase 2 study conducted at FAH-FMU. Eligible patients received SMTR regimen over six 21-day cycles. On Day 0, sintilimab (200 mg intravenously) and rituximab (375 mg/m^2^ intravenously) were administered. MTX was administered intravenously over 4 h on Day 1 at a dose of 3.0 g/m^2^ for patients younger than 65 years. For patients aged 65 years and older, the methotrexate dose was reduced to 1.0 g/m^2^. This adjustment was made due to concerns about the potential for increased treatment-related toxicity and the higher incidence of renal impairment in this older patient population, which can complicate the clearance of methotrexate. Temozolomide (150 mg/m^2^/day orally) was administered on Days 1–5 of each cycle. Each methotrexate dose was followed 24 hours later by leucovorin (30 mg/m^2^), and leucovorin rescue was carried out every 6 h until the blood concentration of methotrexate was less than 0.1 μmol/L.

To evaluate the safety of combination, a safety run-in phase was conducted with the initial six patients. These patients were treated and monitored for DLTs during the 21-day period following the onset of the first treatment cycle. The study definition of DLT is provided in the study protocol (Additional information). If ≤1 patient experienced DLT, enrollment continued according to Simon’s Optimal two-stage design. Otherwise, dose modifications would be considered before proceeding. Participants failing to achieve PR after 4 cycles, or CR after 6 cycles, or experiencing disease progression were taken off from the study. Intrathecal chemotherapy was not administered during treatment.

### Outcomes

Our aim was to assess the efficacy and safety of SMTR regimen for patients with newly diagnosed PCNSL. The primary outcome was the ORR, assessed per the International Primary Central Nervous System Lymphoma Collaborative Group (IPCG) criteria,^43^ which included MRI changes in tumor size, corticosteroid use, ocular examination, and CSF analysis. In our study, gadolinium-enhanced MRI was utilized to assess changes in lesion characteristics. The evaluation focused on the size of enhanced lesions as observed on T1-weighted MRI images. All imaging data were independently measured and evaluated by two radiologists. In instances of disagreement between the two radiologists, a more senior radiology expert was consulted to review the images and achieve consensus. CSF cytology examinations were conducted before treatment, after the fourth course of chemotherapy, and upon completion of treatment. Secondary outcomes were the DOR, PFS, and OS. Beyond the response defined by the IPCG, efficacy was also defined as DCB, comprising CR, PR, or SD that lasted more than 6 months. On the other hand, NDB included patients with PD or SD lasted 6 months or less.^44^ The Common Terminology Criteria for Adverse Events (version 4.0) was used to assess and grade AEs. The study also included exploratory analyses to investigate potential biomarkers of the clinical efficacy of the SMTR regimen.

### Correlative studies

PD-L1 immunostaining was conducted on formalin-fixed, paraffin-embedded tissue sections using primary antibody clone 28-8 (Abcam, Cambridge, UK) according to the recommendations of the manufacturer, as previously described.^45^ Lumbar punctures for CSF samples were performed when feasible. The concentrations of 12 cytokines (IL1β, IL2, IL4, IL5, IL6, IL8, IL10, IL12P70, IL17, TNF, IFNα, and IFNγ) were quantified by flow cytometry using a microsphere-based multiplex immunofluorescence assay kit provided by Qingdao Raisecare Biotechnology Co., Ltd, Qingdao, China.^26^

### WES and mutation analysis

We conducted comprehensive genomic profiling using WES on paired samples obtained from 26 patients, which included formalin-fixed paraffin-embedded (FFPE) tissue and peripheral blood specimens collected before treatment. Somatic mutations, including single nucleotide variants (SNVs) and insertions/deletions (INDELs), were identified using statistical analyses in both tumor and matched peripheral blood samples, with the latter serving as germline controls. Detailed information on the sequencing assays and informatics pipeline can be found in the Supplementary file 1. Further analysis and annotation of the genetic mutations were performed using the maftools R package, and signaling pathway analysis was conducted to elucidate the functional implications of mutations.

### Statistical analysis

The primary outcome, ORR, was summarized with a 95% CI using the Clopper-Pearson method. Our study was designed using Simon’s Optimal two-stage aiming to increase ORR from the 58% reported previously^9^ to >83% with a one-sided α error of 0.05 and a power of 0.8. Initially, 8 eligible patients were enrolled. If >5 patients achieved a PR or better, the trial proceeded to the second stage, enrolling an additional 19 patients. Among 27 evaluable patients, if >19 achieved a PR or better, the SMTR regimen would be considered “promising”. Efficacy analyses were conducted in patients who received at least one cycle of SMTR and undergo at least one post-baseline assessment. Safety analyses were conducted using data from the entire treated analysis set. Data cut-off was of 7 October 2023. DOR, PFS, and OS were estimated using the Kaplan-Meier method. Median values were accompanied by 95% confidence intervals (CIs) calculated using the Brookmeyer-Crowley method. The AUC of the ROC curve was calculated to assess predictive performance. Data analyses were performed using R software (version 4.1.0). P < 0.05 (two-sided) indicated significance.

## Supplementary information

Supplementary file 1

Figure S1

Figure S2

PCNSL Protocol

## Data Availability

The raw sequence data reported in this paper have been deposited in the Genome Sequence Archive in National Genomics Data Center, China National Center for Bioinformation / Beijing Institute of Genomics, Chinese Academy of Sciences (GSA-Human: HRA007993) that are publicly accessible at https://ngdc.cncb.ac.cn/gsa-human.

## References

[1] Fallah, J., Qunaj, L. & Olszewski, A. J. Therapy and outcomes of primary central nervous system lymphoma in the United States: analysis of the National Cancer Database. *Blood Adv.* **1**, 112–121 (2016). 29296804 10.1182/bloodadvances.2016000927 PMC5737162

[2] Houillier, C. et al. Management and outcome of primary CNS lymphoma in the modern era: An LOC network study. *Neurology* **94**, e1027–e1039 (2020). 31907289 10.1212/WNL.0000000000008900 PMC7238921

[3] Illerhaus, G. et al. High-dose chemotherapy with autologous haemopoietic stem cell transplantation for newly diagnosed primary CNS lymphoma: a prospective, single-arm, phase 2 trial. *Lancet Haematol.* **3**, e388–e397 (2016). 27476790 10.1016/S2352-3026(16)30050-3

[4] Schaff, L. R. & Grommes, C. Primary central nervous system lymphoma. *Blood* **140**, 971–979 (2022). 34699590 10.1182/blood.2020008377 PMC9437714

[5] Rubenstein, J. L. et al. Intensive chemotherapy and immunotherapy in patients with newly diagnosed primary CNS lymphoma: CALGB 50202 (Alliance 50202). *J. Clin. Oncol.* **31**, 3061–3068 (2013). 23569323 10.1200/JCO.2012.46.9957 PMC3753699

[6] Morris, P. G. et al. Rituximab, methotrexate, procarbazine, and vincristine followed by consolidation reduced-dose whole-brain radiotherapy and cytarabine in newly diagnosed primary CNS lymphoma: final results and long-term outcome. *J. Clin. Oncol.* **31**, 3971–3979 (2013). 24101038 10.1200/JCO.2013.50.4910 PMC5569679

[7] Ferreri, A. J. M. et al. Chemoimmunotherapy with methotrexate, cytarabine, thiotepa, and rituximab (MATRix regimen) in patients with primary CNS lymphoma: results of the first randomisation of the International Extranodal Lymphoma Study Group-32 (IELSG32) phase 2 trial. *Lancet Haematol.* **3**, e217–e227 (2016). 27132696 10.1016/S2352-3026(16)00036-3

[8] Bromberg, J. E. C. et al. Rituximab in patients with primary CNS lymphoma (HOVON 105/ALLG NHL 24): a randomised, open-label, phase 3 intergroup study. *Lancet Oncol.* **20**, 216–228 (2019). 30630772 10.1016/S1470-2045(18)30747-2

[9] Wieduwilt, M. J. et al. Immunochemotherapy with intensive consolidation for primary CNS lymphoma: a pilot study and prognostic assessment by diffusion-weighted MRI. *Clin. Cancer Res* **18**, 1146–1155 (2012). 22228634 10.1158/1078-0432.CCR-11-0625 PMC3288204

[10] Birsen, R. et al. Efficacy and safety of high-dose etoposide cytarabine as consolidation following rituximab methotrexate temozolomide induction in newly diagnosed primary central nervous system lymphoma in immunocompetent patients. *Haematologica* **103**, e296–e299 (2018). 29472354 10.3324/haematol.2017.185843 PMC6029551

[11] Nastoupil, L. J. et al. Safety and activity of pembrolizumab in combination with rituximab in relapsed or refractory follicular lymphoma. *Blood Adv.* **6**, 1143–1151 (2022). 35015819 10.1182/bloodadvances.2021006240 PMC8864656

[12] Westin, J. R. et al. Safety and activity of PD1 blockade by pidilizumab in combination with rituximab in patients with relapsed follicular lymphoma: a single group, open-label, phase 2 trial. *Lancet Oncol.* **15**, 69–77 (2014). 24332512 10.1016/S1470-2045(13)70551-5 PMC3922714

[13] Wang, S. et al. Temozolomide promotes immune escape of GBM cells via upregulating PD-L1. *Am. J. Cancer Res* **9**, 1161–1171 (2019). 31285949 PMC6610056

[14] Dai, B., Qi, N., Li, J. & Zhang, G. Temozolomide combined with PD-1 Antibody therapy for mouse orthotopic glioma model. *Biochem Biophys. Res Commun.* **501**, 871–876 (2018). 29758196 10.1016/j.bbrc.2018.05.064

[15] Chapuy, B. et al. Targetable genetic features of primary testicular and primary central nervous system lymphomas. *Blood* **127**, 869–881 (2016). 26702065 10.1182/blood-2015-10-673236 PMC4760091

[16] Four, M. et al. PD1 and PDL1 expression in primary central nervous system diffuse large B-cell lymphoma are frequent and expression of PD1 predicts poor survival. *Hematol. Oncol.* **35**, 487–496 (2017). 27966264 10.1002/hon.2375

[17] Qiu, Y. et al. Immune checkpoint inhibition by anti-PDCD1 (anti-PD1) monoclonal antibody has significant therapeutic activity against central nervous system lymphoma in an immunocompetent preclinical model. *Br. J. Haematol.* **183**, 674–678 (2018). 29076134 10.1111/bjh.15009

[18] Nayak, L. et al. PD-1 blockade with nivolumab in relapsed/refractory primary central nervous system and testicular lymphoma. *Blood* **129**, 3071–3073 (2017). 28356247 10.1182/blood-2017-01-764209 PMC5766844

[19] Graber, J. J., Plato, B., Mawad, R. & Moore, D. J. Pembrolizumab immunotherapy for relapsed CNS Lymphoma. *Leuk. Lymphoma* **61**, 1766–1768 (2020). 32204633 10.1080/10428194.2020.1742903

[20] Gavrilenko, A. et al. PB2308: NIVOLUMAB-BASED THERAPY OF RELAPSED OR REFRACTORY PRIMARY LARGE B-CELL LYMPHOMA OF IMMUNE-PRIVILEGED SITES AND DLBCL WITH SECONDARY CNS INVOLVEMENT. *Hemasphere* **7**, e49127b4 (2023). 10.1097/01.HS9.0000975956.49127.b4

[21] Zhang, L., Mai, W., Jiang, W. & Geng, Q. Sintilimab: A Promising Anti-Tumor PD-1 Antibody. *Front Oncol.* **10**, 594558 (2020). 33324564 10.3389/fonc.2020.594558 PMC7726413

[22] Cho, H. et al. Programmed cell death 1 expression is associated with inferior survival in patients with primary central nervous system lymphoma. *Oncotarget* **8**, 87317–87328 (2017). 29152083 10.18632/oncotarget.20264 PMC5675635

[23] Villanueva, G. et al. A Systematic Review of High-Dose Methotrexate for Adults with Primary Central Nervous System Lymphoma. *Cancers (Basel)* **15**, 1459 (2023). 36900250 10.3390/cancers15051459 PMC10000886

[24] Dalia, S., Price, S., Forsyth, P., Sokol, L. & Jaglal, M. What is the optimal dose of high-dose methotrexate in the initial treatment of primary central nervous system lymphoma? *Leuk. Lymphoma* **56**, 500–502 (2015). 24882264 10.3109/10428194.2014.927458

[25] Kasenda, B. et al. First-line treatment and outcome of elderly patients with primary central nervous system lymphoma (PCNSL)-a systematic review and individual patient data meta-analysis. *Ann. Oncol.* **26**, 1305–1313 (2015). 25701456 10.1093/annonc/mdv076 PMC4735103

[26] Chinese Society of Clinical Oncology (CSCO). Anti‐leukemia Alliance of CSCO, Anti‐lymphoma Alliance of CSCO [Experts consensus on high‐dose methotrexate and calcium folinate rescue therapy in the treatment of malignant tumors]. *Chin. J. Clin. Oncol*. 46, 761–767 (2019).

[27] Chen, T. et al. Evidence-based expert consensus on the management of primary central nervous system lymphoma in China. *J. Hematol. Oncol.* **15**, 136 (2022). 36176002 10.1186/s13045-022-01356-7 PMC9524012

[28] Illerhaus, G. et al. Effects on Survival of Non-Myeloablative Chemoimmunotherapy Compared to High-Dose Chemotherapy Followed By Autologous Stem Cell Transplantation (HDC-ASCT) As Consolidation Therapy in Patients with Primary CNS Lymphoma - Results of an International Randomized Phase III Trial (MATRix/IELSG43). *Blood* **140**, LBA-3 (2022). 10.1182/blood-2022-171733

[29] Houillier, C. et al. Radiotherapy or Autologous Stem-Cell Transplantation for Primary CNS Lymphoma in Patients Age 60 Years and Younger: Long-Term Results of the Randomized Phase II PRECIS Study. *J. Clin. Oncol.* **40**, 3692–3698 (2022). 35834762 10.1200/JCO.22.00491

[30] Scordo, M. et al. Outcomes Associated With Thiotepa-Based Conditioning in Patients With Primary Central Nervous System Lymphoma After Autologous Hematopoietic Cell Transplant. *JAMA Oncol.* **7**, 993–1003 (2021). 33956047 10.1001/jamaoncol.2021.1074 PMC8283558

[31] Ferreri, A. J. M. et al. Long-term efficacy, safety and neurotolerability of MATRix regimen followed by autologous transplant in primary CNS lymphoma: 7-year results of the IELSG32 randomized trial. *Leukemia* **36**, 1870–1878 (2022). 35562406 10.1038/s41375-022-01582-5

[32] Batchelor, T. T. et al. Myeloablative vs nonmyeloablative consolidation for primary central nervous system lymphoma: results of Alliance 51101. *Blood Adv.* **8**, 3189–3199 (2024). 38598710 10.1182/bloodadvances.2023011657 PMC11225669

[33] Schorb, E. et al. High-dose chemotherapy and autologous haematopoietic stem-cell transplantation in older, fit patients with primary diffuse large B-cell CNS lymphoma (MARTA): a single-arm, phase 2 trial. *Lancet Haematol.* **11**, e196–e205 (2024). 38301670 10.1016/S2352-3026(23)00371-X

[34] Morales-Martinez, A. et al. Prognostic factors in primary central nervous system lymphoma. *Curr. Opin. Oncol.* **34**, 676–684 (2022). 36093869 10.1097/CCO.0000000000000896

[35] Grommes, C. Circulating Tumor DNA in the Blood: A New Frontier in Primary CNS Lymphoma? *J. Clin. Oncol.* **41**, 1649–1651 (2023). 36669147 10.1200/JCO.22.02605 PMC10043552

[36] Glass, J. et al. Phase I and II Study of Induction Chemotherapy With Methotrexate, Rituximab, and Temozolomide, Followed By Whole-Brain Radiotherapy and Postirradiation Temozolomide for Primary CNS Lymphoma: NRG Oncology RTOG 0227. *J. Clin. Oncol.* **34**, 1620–1625 (2016). 27022122 10.1200/JCO.2015.64.8634 PMC4872318

[37] Tang, Q. et al. The role of PD-1/PD-L1 and application of immune-checkpoint inhibitors in human cancers. *Front Immunol.* **13**, 964442 (2022). 36177034 10.3389/fimmu.2022.964442 PMC9513184

[38] Ma, H. et al. Interferon-alpha promotes immunosuppression through IFNAR1/STAT1 signalling in head and neck squamous cell carcinoma. *Br. J. Cancer* **120**, 317–330 (2019). 30555157 10.1038/s41416-018-0352-y PMC6353953

[39] Cheon, H., Wang, Y., Wightman, S. M., Jackson, M. W. & Stark, G. R. How cancer cells make and respond to interferon-I. *Trends Cancer* **9**, 83–92 (2023). 36216730 10.1016/j.trecan.2022.09.003 PMC9797472

[40] Takashima, Y. et al. Target amplicon exome-sequencing identifies promising diagnosis and prognostic markers involved in RTK-RAS and PI3K-AKT signaling as central oncopathways in primary central nervous system lymphoma. *Oncotarget* **9**, 27471–27486 (2018). 29937999 10.18632/oncotarget.25463 PMC6007945

[41] Han, S. et al. Alterations in the RTK/Ras/PI3K/AKT pathway serve as potential biomarkers for immunotherapy outcome of diffuse gliomas. *Aging (Albany NY)* **13**, 15444–15458 (2021). 34100771 10.18632/aging.203102 PMC8221357

[42] Alame, M. et al. The immune contexture of primary central nervous system diffuse large B cell lymphoma associates with patient survival and specific cell signaling. *Theranostics* **11**, 3565–3579 (2021). 33664848 10.7150/thno.54343 PMC7914352

[43] Abrey, L. E. et al. Report of an international workshop to standardize baseline evaluation and response criteria for primary CNS lymphoma. *J. Clin. Oncol.* **23**, 5034–5043 (2005). 15955902 10.1200/JCO.2005.13.524

[44] Rizvi, N. A. et al. Mutational landscape determines sensitivity to PD-1 blockade in non–small cell lung cancer. *Science* **348**, 124–128 (2015). 25765070 10.1126/science.aaa1348 PMC4993154

[45] Stoy, S. P., Rosen, L., Mueller, J. & Murgu, S. Programmed death-ligand 1 testing of lung cancer cytology specimens obtained with bronchoscopy. *Cancer Cytopathol.* **126**, 122–128 (2018). 29053224 10.1002/cncy.21941

