# Supplementary material for: Sintilimab (anti-PD-1 antibody) combined with high-dose methotrexate, temozolomide, and rituximab (anti-CD20 antibody) in primary central nervous system lymphoma: a phase 2 study
Source: Signal Transduct Target Ther. 2024 Sep 4;9:229. doi: 10.1038/s41392-024-01941-x (PMC11372099; doi:10.1038/s41392-024-01941-x)
Supplement: Supplementary file 1 [file 41392_2024_1941_MOESM1_ESM.docx]

Supplementary Materials for

**Sintilimab (anti-PD-1 antibody) combined with high-dose methotrexate, temozolomide, and rituximab (anti-CD20 antibody) in primary central nervous system lymphoma: a phase 2 study**

Zhiyong Zeng, Apeng Yang, Jingke Yang, Sheng Zhang, Zhen Xing, Xingfu Wang, Wenzhong Mei, Changzhen Jiang, Junfang Lin, Xiyue Wu, Yihui Xue, Zhanyi Wu^6^, Lianghong Yu^6^, Dengliang Wang, Jianwu Chen, Shufa Zheng, Qiaoxian Lin, Qingjiao Chen, Jinfeng Dong, Xiaoqiang Zheng, Jizhen Wang, Jinlong Huang, Zhenying Chen, Ping Chen, Meihong Zheng, Xiaofang Zhou, Youwen He, Yuanxiang Lin, Junmin Chen

Correspondence to: Dr. Zhiyong Zeng, Department of Hematology (Email: zengzhiyong049@163.com); or Dr. Yuanxiang Lin, Department of Neurosurgery (Email: lyx99070@163.com); or Dr. Junmin Chen, Department of Hematology (Email: drjunminchen@fjmu.edu.cn). They are all located at the First Affiliated Hospital of Fujian Medical University, Fuzhou, 350005, China.

**This PDF file includes:**

Materials and Methods

Figures. S1 to S2

Tables S1

References

1. Supplementary materials and methods

**Whole-exome sequencing (WES)**

DNA was extracted using the QIAamp DNA Mini Kit. Analysis of tumor-associated mutations demonstrated that average tumor cellularity >50%. All patients also submitted a normal tissue resected at surgery, peripheral blood or buccal swab for germline analysis.

Recovered DNA was quantified by Qubit using standard procedures. Integrity of genomic DNA was confirmed by gel electrophoresis. The exome sequences were efficiently enriched from 400 ng genomic DNA using Agilent SureSelect Human All Exon V6 (Agilent USA, Catalog #: 5190-8864) according to the manufacturer’s protocol. Firstly, qualified genomic DNA was randomly fragmented to an average size of 180-280 bp by Covaris LE220R-plus (Covaris, USA). Remaining overhangs were converted into blunt ends via exonuclease polymerase activities. Secondly, DNA fragments were end repaired and phosphorylated, followed by A-tailing and ligation at the 3’ ends with paired-end adapters. DNA fragments with ligated adapter molecules on both ends were selectively enriched in a PCR reaction. After PCR reaction, libraries hybridize with liquid phase with biotin labeled probe, then use magnetic beads with streptomycin to capture the exons of genes. Captured libraries were enriched in a PCR reaction to add index tags to prepare for sequencing. Products were purified using AMPure XP system (Beckman Coulter, Beverly, USA), libraries were analyzed for size distribution by Agilent 5400 system (AATI) (Agilent, USA) and quantified by real-time PCR (Life Technologies, USA) (1.5 nM). The qualified libraries were pooled and sequenced on NovaSeq 6000 system with PE150 strategy in Shanghai Tissuebank Co., Ltd (China), according to effective library concentration and the data amount required.

The sequence reads were aligned to the human genome (Build-UCSC hg19) using the BWA alignment algorithm. SAMtools was used to call germline SNP and InDel.^1^ The filter parameters of SNP and InDel are shown as follows: QUAL≥20; DP≥4; MQ≥30. Somatic mutation detection is commonly applied to normal and tumor- paired samples. The somatic SNV was detected by MuTect,^2^ while the somatic InDel was identified by Strelka.^3^ Control-FREEC was used to detect somatic CNV.^4^ The parameter with window = 0 was set as officially recommended in the config file for somatic CNV detection. ANNOVAR was used to perform variant annotation.^5^ Annotation contents refer to protein-coding changes, genomic regions affected by the variants, allele frequency, deleterious prediction, etc.

A general sequencing depth of 200× was observed for sequenced cases, and represented >99.00% of the target sequences. With the deeper sequencing, relatively few additional mutations were discovered. The increased depth largely served to detect mutations with low sequence coverage and allele frequency. Minimum of 14 reads covering a site in the tumor and 8 in the normal were required for mutation calling. Only INDELS with reference allele counts greater than eight and tumor variant allele counts greater than three were considered.

1. Supplementary Figures

**Figure. S1. PFS of patients with PCNSL according to clinical traits.**

**
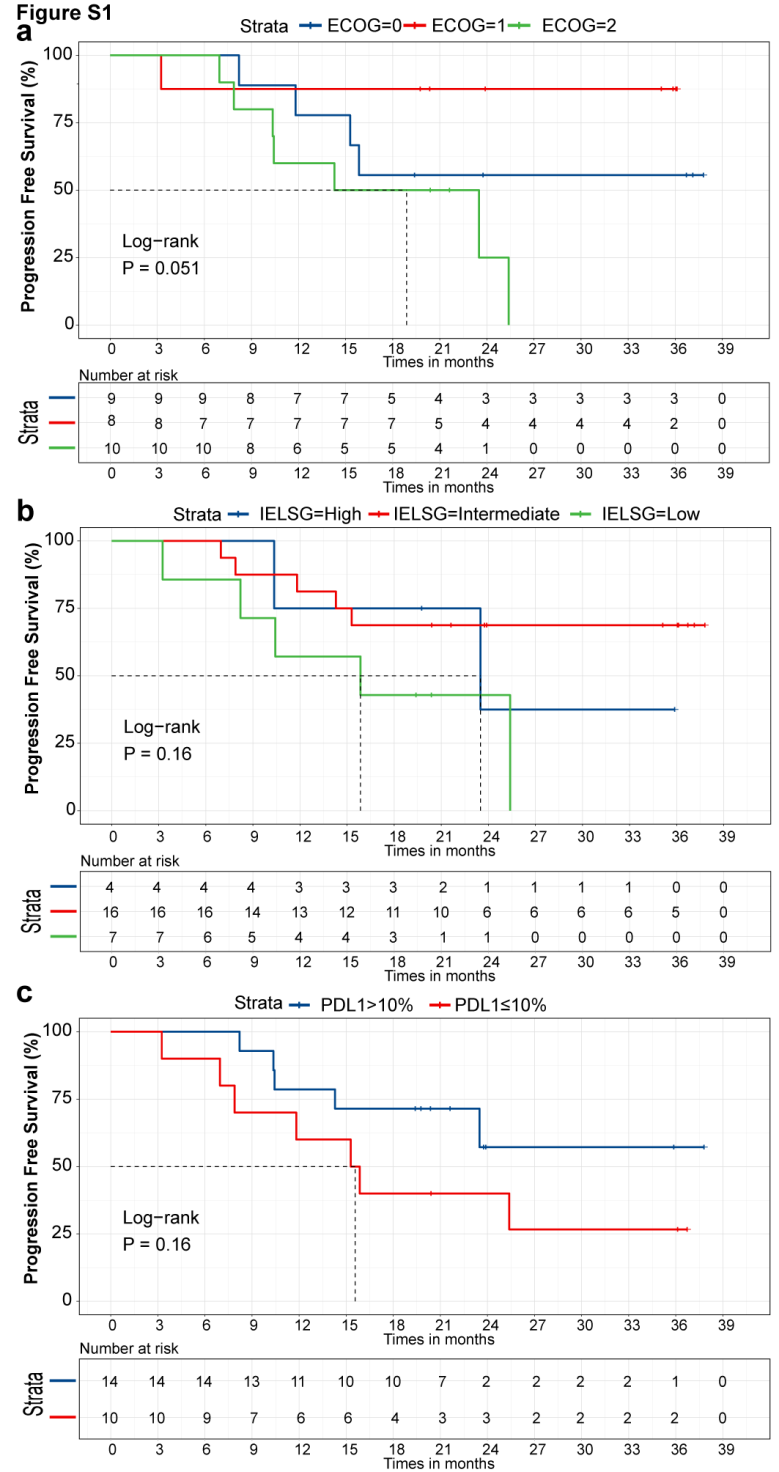
**

PFS was analyzed according to the following parameters at baseline: panel **a**, ECOG PS; panel **b**, IELSG risk; panel **C**, PD-L1 expression of tumor cells. The optimal cut-off point of PD-L1 expression as determined by ROC analysis were calculated and was defined as the threshold. ECOG PS, Eastern Cooperative Oncology Group performance status; IELSG, International Extranodal Lymphoma Study Group; PCNSL, primary central nervous system lymphoma; PD-L1, programmed death-ligand 1; PFS, progression free survival; ROC, receiver operating characteristic.

**Figure. S2. PFS of patients with PCNSL according to the cytokine concentration in CSF**


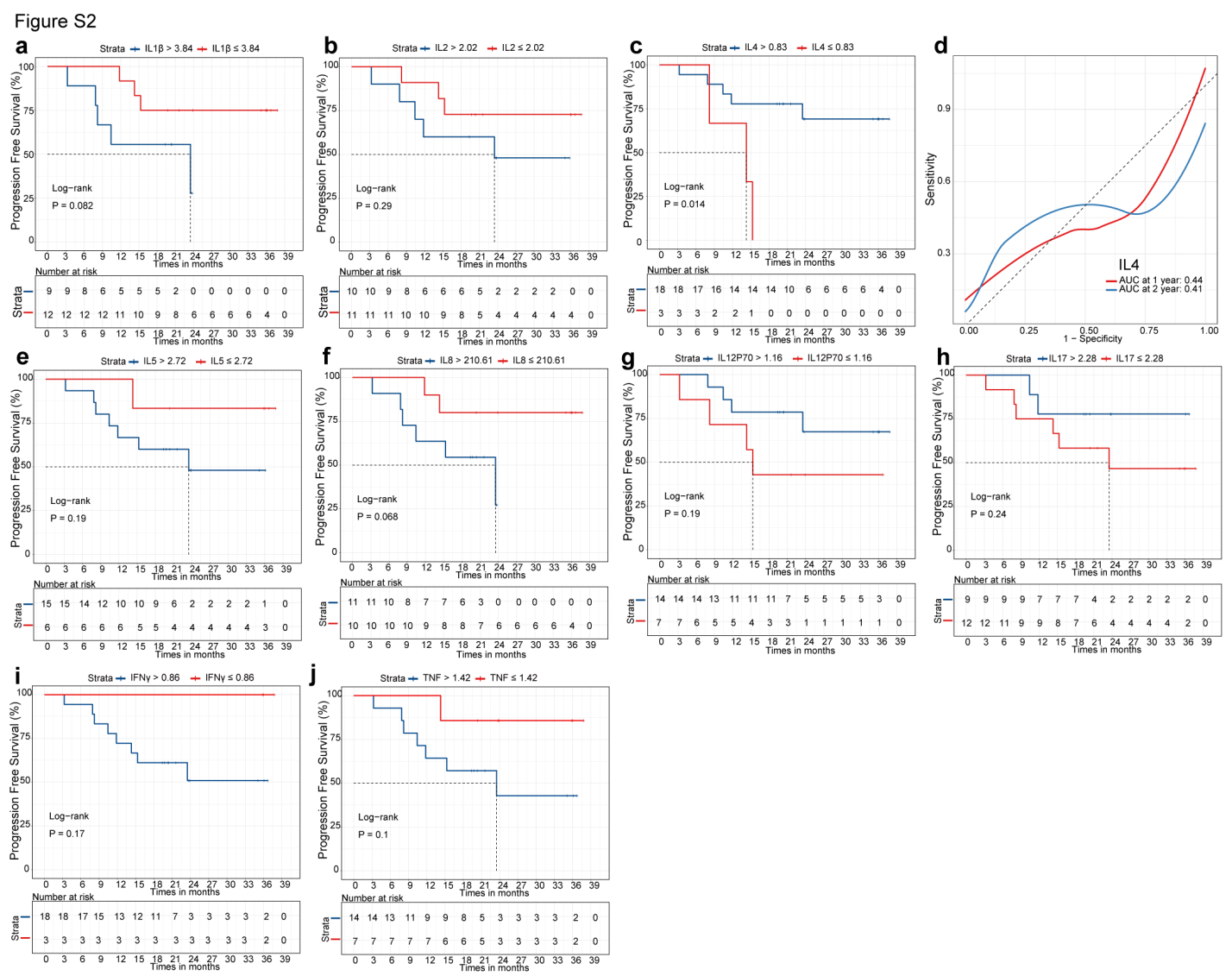


PFS of patients with PCNSL according to the cytokine concentration in CSF. The optimal cut-off point of cytokines as determined by ROC analysis were calculated and was defined as the threshold. PFS was analyzed according to the following cytokine concentrations in CSF at baseline: panel **a**, IL1β; panel **b** IL2; panel **c**, IL4; panel **d**, Time-dependent ROC analysis of predicting PFS by IL4; panel **e**, IL5; panel **f**, IL8; panel **g**, IL12P70; panel **h**, IL17; panel **i**, IFNγ; panel **j**, TNF. CSF, cerebrospinal fluid; IL1β, interleukin 1β; IL2, interleukin 2; IL4, interleukin 4; IL5, interleukin 5; IL8, interleukin 8; IL12P70, interleukin 12P70; IL17, interleukin 17; IFNγ, interferon-γ; PCNSL, primary central nervous system lymphoma; PFS, progression free survival; ROC, receiver operating characteristic; TNF, tumor necrosis factor.

1. Supplementary Tables

Table S1. Patient’s methotrexate administration and steroid use

| Patient ID | Methotrexate dose (g) | | | | | | | Steroid use because of immune-related adverse events |
| --- | --- | --- | --- | --- | --- | --- | --- | --- |
|  | Cycle 1 | Cycle 2 | Cycle 3 | Cycle 4 | Cycle 5 | Cycle 6 | Cumulative total dose |  |
| 1 | 4.74 | 4.74 | 4.74 | 4.74 | 4.74 | 4.74 | 28.44 | / |
| 2 | 5.1 | 5.1 | 5.1 | 5.1 | 5.1 | 5.1 | 30.6 | / |
| 3 | 1.6 | 1.6 | 1.6 | 1.6 | 1.6 | 1.6 | 9.6 | / |
| 4 | 5.29 | 5.29 | 5.29 | 5.29 | 5.29 | 5.29 | 31.74 | / |
| 5 | 4.5 | 4.5 | 4.5 | 4.5 | 4.5 | 4.5 | 27 | After the second cycle of chemotherapy, rash appeared and methylprednisolone 8 mg was administered, which was discontinued 7 days later. |
| 6 | 5.6 | 5.6 | 5.6 | 5.6 | 5.6 | 5.6 | 33.6 | / |
| 7 | 4.8 | 4.8 | 4.8 | 4.8 | 4.8 | 4.8 | 28.8 | / |
| 8 | 4.35 | 4.35 | 4.35 | 4.35 | 4.35 | 4.35 | 26.1 | / |
| 9 | 5.1 | 5.1 | 5.1 | 5.1 | 5.1 | 5.1 | 30.6 | / |
| 10 | 5.04 | 5.04 | 5.04 | 5.04 | 5.04 | 5.04 | 30.24 | / |
| 11 | 4.11 | 4 | 4 | 4 | 4 | 4 | 24.11 | / |
| 12 | 1.64 | 1.64 | 1.64 | 1.64 | 1.64 | /$ | 8.2 | / |
| 13 | 4.38 | 4.38 | 4.38 | 4.38 | 4.38 | 4.38 | 26.28 | / |
| 14 | 5.49 | 5.49 | 5.49 | 5.49 | 5.49 | 5.49 | 32.94 | Interstitial pneumonia and fever occurred during the third cycle of chemotherapy, and methylprednisolone was given 60 mg qd, which was reduced to 20 mg qd after 10 days, then to 8 mg qd after 6 days, and was discontinued after 5 days. |
| 15 | 5.4 | 5.4 | 5.4 | 5.4 | 5.4 | 5.4 | 32.4 | / |
| 16 | 4.4 | 4.4 | 4.4 | 4.4 | 4.4 | 4.4 | 26.4 | / |
| 17 | 4.98 | 4.98 | 4.98 | 4.98 | 4.98 | 4.98 | 29.88 | / |
| 18 | 4.62 | 4.62 | 4.62 | 4.62 | 4.62 | 4.62 | 27.72 | / |
| 19 | 4.41 | 4.41 | 4.41 | 4.41 | /* | /* | 17.64 | / |
| 20 | 5.19 | 5.19 | 5.19 | 5.19 | 5.19 | 5.19 | 31.14 | / |
| 21 | 5.34 | 5.34 | 5.34 | 5.34 | 5.34 | /# | 26.7 | / |
| 22 | 5.58 | 5.58 | 5.58 | 5.58 | 5.67 | 5.67 | 33.66 | / |
| 23 | 4.68 | 4.68 | 4.68 | 4.68 | 4.68 | 4.68 | 28.08 | / |
| 24 | 4.2 | 4.2 | 4.2 | 4.2 | 4.2 | 4.2 | 25.2 | / |
| 25 | 4.38 | 4.38 | 4.38 | 4.38 | 4.38 | 4.38 | 26.28 | Interstitial pneumonia occurred during the second cycle of chemotherapy, and methylprednisolone 20 mg was given. After 7 days, it was reduced to 12 mg qd, then to 4 mg qd after 7 days, and was discontinued after 7 days. |
| 26 | 5.97 | 5.97 | /* | /* | /* | /* | 11.94 | During the second cycle of chemotherapy, a rash appeared and prednisone was given 40 mg qd, which was reduced to 8 mg qd after 3 days, and was discontinued after 3 days. |
| 27 | 4.32 | 4.32 | 4.32 | 4.32 | 4.32 | 4.32 | 25.92 | / |

Note: *Discontinued due to disease progression; $ Voluntarily discontinued treatment after five cycles; #Discontinued after five cycles due to COVID-19 infection .

References

1. Li, H. *et al.* The Sequence Alignment/Map format and SAMtools. *Bioinformatics* **25**, 2078–2079 (2009).

2. Cibulskis, K. *et al.* Sensitive detection of somatic point mutations in impure and heterogeneous cancer samples. *Nat Biotechnol* **31**, 213–219 (2013).

3. Saunders, C. T. *et al.* Strelka: accurate somatic small-variant calling from sequenced tumor-normal sample pairs. *Bioinformatics* **28**, 1811–1817 (2012).

4. Boeva, V. *et al.* Control-FREEC: a tool for assessing copy number and allelic content using next-generation sequencing data. *Bioinformatics* **28**, 423–425 (2012).

5. Wang, K., Li, M. & Hakonarson, H. ANNOVAR: functional annotation of genetic variants from high-throughput sequencing data. *Nucleic Acids Res* **38**, e164 (2010).
