# Supplementary figures and images for: Sintilimab (anti-PD-1 antibody) combined with high-dose methotrexate, temozolomide, and rituximab (anti-CD20 antibody) in primary central nervous system lymphoma: a phase 2 study
Source: Signal Transduct Target Ther. 2024 Sep 4;9:229. doi: 10.1038/s41392-024-01941-x (PMC11372099; doi:10.1038/s41392-024-01941-x)

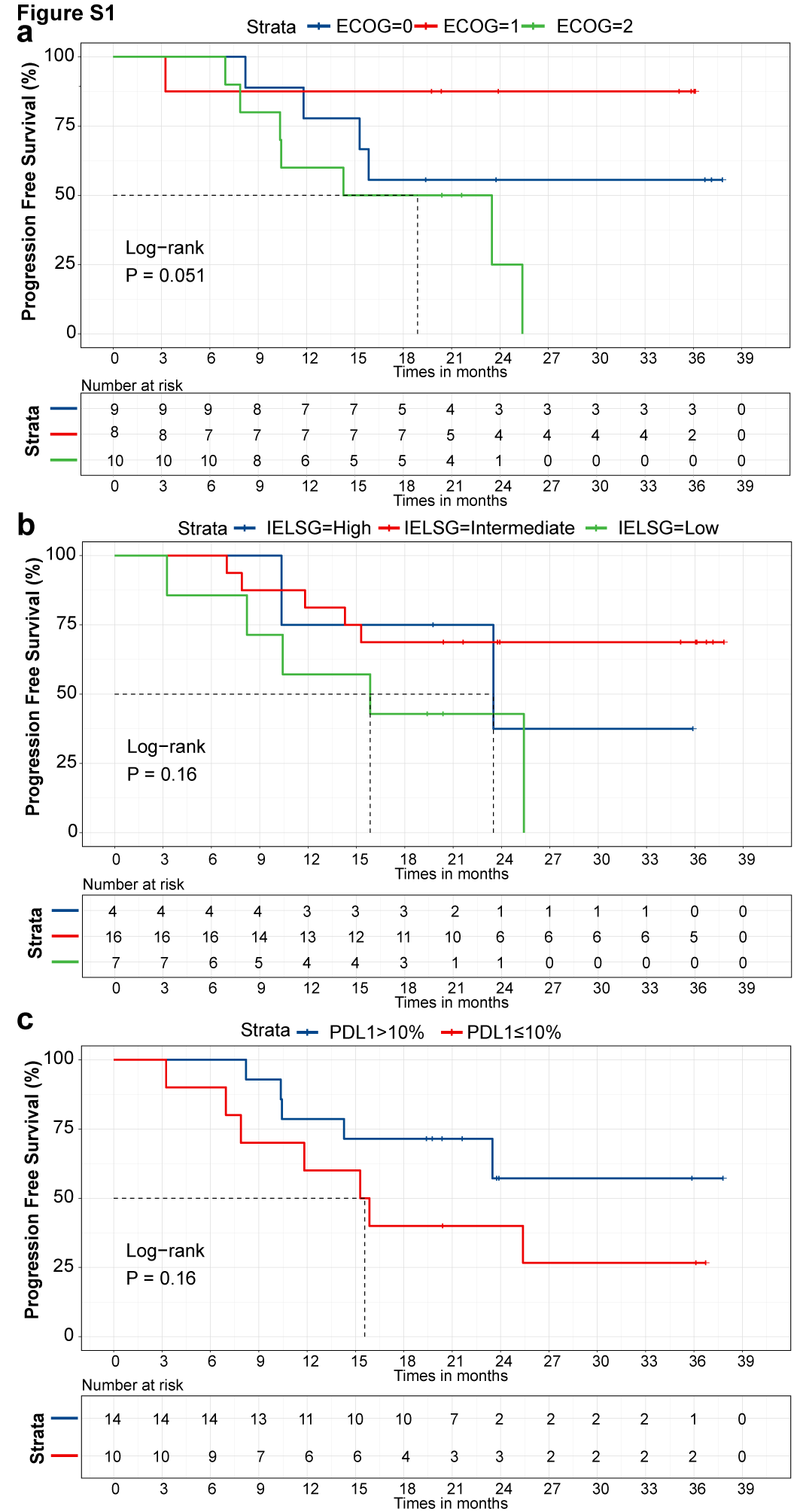

Supplement: Supplementary file 2 — Figure S1 [file 41392_2024_1941_MOESM2_ESM.tif]

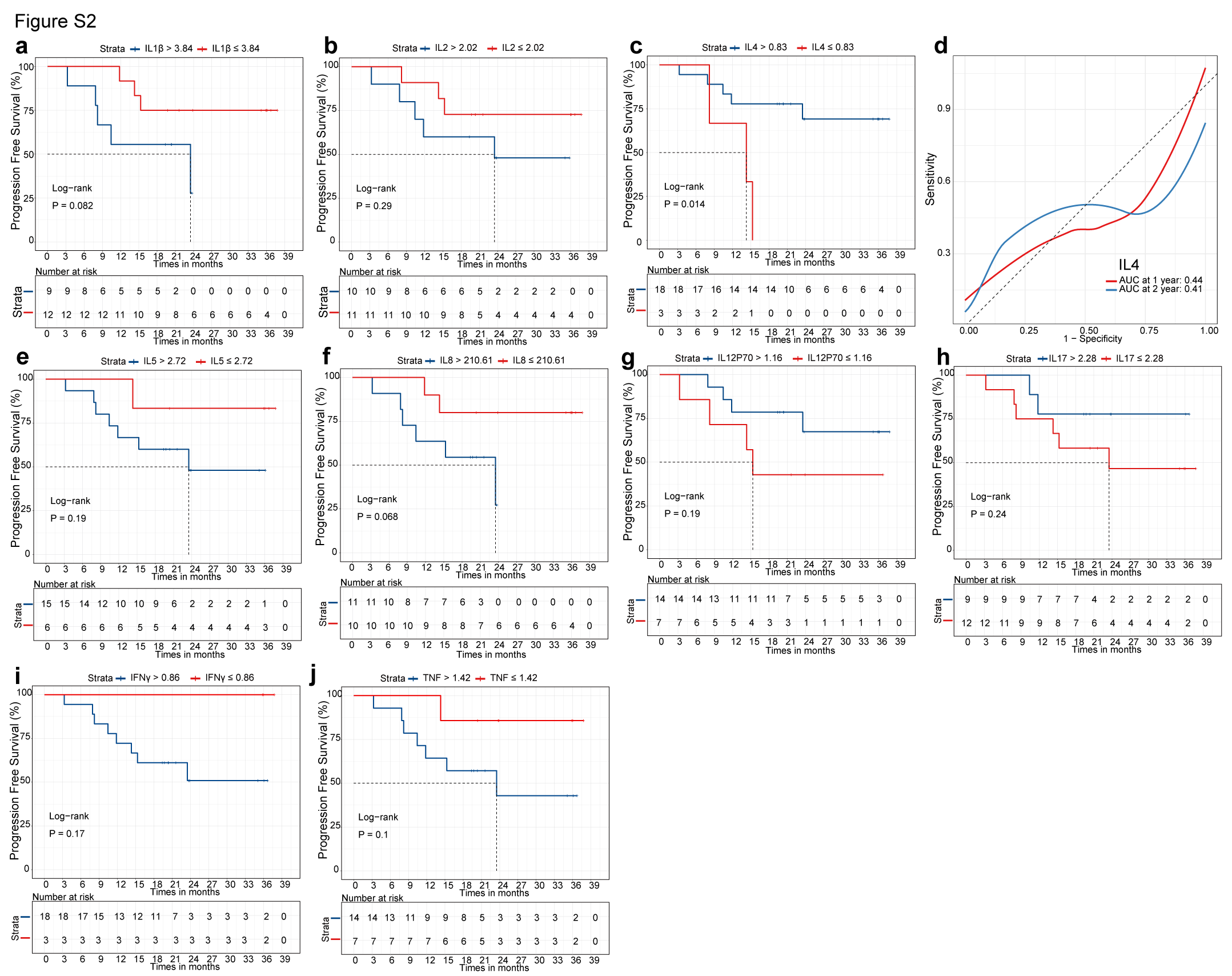

Supplement: Supplementary file 3 — Figure S2 [file 41392_2024_1941_MOESM3_ESM.tif]
