# Supplementary material for: Sintilimab (anti-PD-1 antibody) combined with high-dose methotrexate, temozolomide, and rituximab (anti-CD20 antibody) in primary central nervous system lymphoma: a phase 2 study
Source: Signal Transduct Target Ther. 2024 Sep 4;9:229. doi: 10.1038/s41392-024-01941-x (PMC11372099; doi:10.1038/s41392-024-01941-x)
Supplement: Supplementary file 4 — PCNSL Protocol [file 41392_2024_1941_MOESM4_ESM.doc]

The following protocol information is provided solely to describe how the authors conducted the research underlying the published report associated with the following article:

Study of Frontline Sintilimab plus R-MT for Primary Central Nervous System Diffuse Large B-cell Lymphoma

**List of Abbreviations**

| AE | Adverse event |
| --- | --- |
| ALT | Alanine aminotransferase |
| AST | Aspartate aminotransferase |
| CI | Confidence interval |
| CR | Complete response |
| CRF | Case Report Form |
| CSF | Cerebrospinal fluid |
| CT | Computed tomography |
| CTCAE | ommon Terminology Criteria for Adverse Events |
| ctDNA | Circulating tumor DNA |
| DLBCL | Diffuse large B-cell lymphoma |
| DLT | dose limiting toxicity |
| ECOG | Eastern Cancer Cooperation Group |
| HIV | Human Immunodeficiency Virus |
| MMSE | Mini-Mental State Examination |
| MR | Minimal response |
| MRI | Magnetic resonance imaging |
| MTX | methotrexate |
| ORR | Overall response rate |
| OS | Overall survival |
| PCNSL | Primary central nervous system lymphoma |
| PD | Progressive disease |
| PD-1 | Programmed cell death-1 |
| PD-L1 | Programmed cell death-ligand 1 |
| PFS | Progress free survival |
| PR | Partial response |
| SAE | Serious adverse event |
| SD | Stable disease |
| TMZ | Temozolamide |
| ULN | Upper normal limit |

**Table of Contents**

[Title page 1](#__RefHeading___Toc2075161668)

[1 SYNOPSIS 2](#__RefHeading___Toc2107726796)

[2 BACKGROUND AND RATIONALE 4](#__RefHeading___Toc1821503107)

[2.1 Overview of Disease 4](#__RefHeading___Toc1623331364)

[2.2 Current “standard” treatment 5](#__RefHeading___Toc1697983260)

[2.3 Methotrexate 6](#__RefHeading___Toc94465837)

[2.4 Temozolomide 7](#__RefHeading___Toc696907326)

[2.5 Rituximab 8](#__RefHeading___Toc545617344)

[2.6 PD-1 Inhibitors 9](#__RefHeading___Toc435527918)

[2.7 Study Rationale 10](#__RefHeading___Toc1293448850)

[3 OBJECTIVES AND ENDPOINTS 12](#__RefHeading___Toc17863369)

[3.1 Primary Objective 12](#__RefHeading___Toc1729415850)

[3.2 Secondary Objectives 12](#__RefHeading___Toc101028805)

[3.3 Exploratory Objectives 13](#__RefHeading___Toc1479044505)

[4 OVERVIEW OF STUDY DESIGN/INTERVENTION 14](#__RefHeading___Toc1177781510)

[4.1 Study Design 14](#__RefHeading___Toc1617064171)

[4.2 Intervention 15](#__RefHeading___Toc1591969212)

[4.2.1 Dosing and Schedule of Treatment 15](#__RefHeading___Toc727788111)

[4.2.2 Safety Window and DLT definition 15](#__RefHeading___Toc2015411912)

[4.2.3 Safety Monitoring Beyond After Completion of Safety Window 16](#__RefHeading___Toc768440853)

[5 SELECTION, WITHDRAWAL, AND REPLACEMENT OF SUBJECTS 17](#__RefHeading___Toc218763313)

[5.1 SUBJECT POPULATION 17](#__RefHeading___Toc262997927)

[5.1.1 Inclusion Criteria 17](#__RefHeading___Toc684813563)

[5.1.2 Exclusion criteria 18](#__RefHeading___Toc1296689068)

[5.2 Premature Withdrawal from the Study or from Study Treatment 19](#__RefHeading___Toc789116120)

[5.2.1 Reasons for Premature Withdrawal or Discontinuation of Study Treatment 19](#__RefHeading___Toc1963108615)

[5.2.2 Withdrawal from Study Participation 19](#__RefHeading___Toc27739797)

[5.2.3 Discontinuation of Study Treatment 20](#__RefHeading___Toc218816780)

[5.3 Replacement of Patients 21](#__RefHeading___Toc1161617796)

[6 THERAPEUTIC/DIAGNOSTIC AGENTS 21](#__RefHeading___Toc536462495)

[6.1 Sintilimab 21](#__RefHeading___Toc1188803359)

[6.1.1 General Information 21](#__RefHeading___Toc30203025)

[6.1.2 Formulation 21](#__RefHeading___Toc816100483)

[6.1.3 Clinical Pharmacology 22](#__RefHeading___Toc222764392)

[6.1.4 Pharmacokinetics and Drug Metabolism 22](#__RefHeading___Toc937139623)

[6.1.5 Supplier 23](#__RefHeading___Toc860576663)

[6.2 Rituximab 23](#__RefHeading___Toc409612496)

[6.3 Methotrexate 23](#__RefHeading___Toc1672131637)

[6.4 Temozolomide 23](#__RefHeading___Toc1545418417)

[7 INTERVENTION ALLOCATION AND BLINDING 24](#__RefHeading___Toc32624054)

[8 EVALUATION PRIOR TO TREATMENT 24](#__RefHeading___Toc704145593)

[9 TREATMENT/INTERVENTION PLAN 25](#__RefHeading___Toc1940086581)

[10 EVALUATION DURING TREATMENT/INTERVENTION 26](#__RefHeading___Toc1790954466)

[10.1 Safety Evaluation 30](#__RefHeading___Toc1440913710)

[10.2 Adverse Events and Serious Adverse Events 30](#__RefHeading___Toc263636751)

[10.2.1 Adverse Events Definitions 30](#__RefHeading___Toc684110296)

[10.2.2 Serious Adverse Event 31](#__RefHeading___Toc214298834)

[10.2.3 Causality 32](#__RefHeading___Toc390427019)

[10.2.4 Adverse Events Reporting Procedures 33](#__RefHeading___Toc1344366748)

[10.3 Expected Side Effects and Supportive Care for Sintilimab and RMT 34](#__RefHeading___Toc1096483549)

[10.3.1 Dose Modification and Toxicity Management for Immune-related AEs Associated with Sintilimab 34](#__RefHeading___Toc1041833136)

[10.3.2 Dose Modification and Toxicity Management of Infusion-reactions Related to Sintilimab 38](#__RefHeading___Toc1655342761)

[10.3.3 Dose Delay or Modification of Rituximab, HD-MTX and Temozolamide 40](#__RefHeading___Toc695137242)

[10.3.4 Treatment Discontinuation Criteria 42](#__RefHeading___Toc860586614)

[10.3.4.1 Sintilimab 42](#__RefHeading___Toc576858953)

[10.3.4.2 Rituximab 42](#__RefHeading___Toc1527240513)

[10.3.4.3 HD-MTX and Temozolamide 42](#__RefHeading___Toc1606753047)

[10.3.5 Diet/Contraception/Other Considerations 43](#__RefHeading___Toc91599904)

[10.3.5.1 Diet 43](#__RefHeading___Toc1921295276)

[10.3.5.2 Contraception 43](#__RefHeading___Toc1645587440)

[10.3.5.3 Use in Pregnancy 45](#__RefHeading___Toc2093698014)

[10.3.5.4 Use in Nursing Women 45](#__RefHeading___Toc115481556)

[10.4 Evaluation of Response 45](#__RefHeading___Toc1720778451)

[10.5 Evaluation of Biomarkers 46](#__RefHeading___Toc961151808)

[11 STATISTICAL METHODS 47](#__RefHeading___Toc706444322)

[11.1 Sample Size Determination 47](#__RefHeading___Toc1920119238)

[11.2 Efficacy Analyses 49](#__RefHeading___Toc1207269597)

[11.3 Safety Analyses 49](#__RefHeading___Toc1154619923)

[12 SUPPORTING DOCUMENTS AND PRECAUTIONS 50](#__RefHeading___Toc1034811569)

[12.1 Informed Consent Process 50](#__RefHeading___Toc1755466777)

[12.2 Privacy of Personal Data 50](#__RefHeading___Toc1999778553)

[12.3 Collection and Use of Specimens and Data 51](#__RefHeading___Toc11581074)

[12.4 Quality Control and Quality Assurance 51](#__RefHeading___Toc1369582488)

[12.5 Data Processing and Record Storage 51](#__RefHeading___Toc1843147270)

[12.5.1 Data Collection and Management 51](#__RefHeading___Toc324558915)

[12.5.2 Research Data Retention 51](#__RefHeading___Toc253221025)

[12.6 Statement of Conflict of Interest 52](#__RefHeading___Toc1720662468)

[13 REFERENCES 52](#__RefHeading___Toc1159309174)

[14 APPENDICES 57](#__RefHeading___Toc390158187)

[Appendix 1: ECOG Performance Status 57](#__RefHeading___Toc1121074618)

[Appendix 2: National Cancer Institute Common Terminology Criteria for Adverse Events 57](#__RefHeading___Toc2027069595)

The First Affiliated Hospital of Fujian Medical University

# Title page

Study of Frontline Sintilimab plus R-MT for Primary Central Nervous System Diffuse Large B-cell Lymphoma

| Principal Investigator: | Zhiyong Zeng M.D. Department of Hematology,  The First Affiliated Hospital of Fujian Medical University Fujian 350005, P.R. China |
| --- | --- |
|  |  |
|  |  |
|  |  |
| Co-Investigator: | Jinlong Huang M.D. |
|  | Junmin Chen M.D. |
|  | Junfang Lin B.D. |
|  | Apeng Yang B.D. |
|  | Jinfeng Dong B.D.  Qingjiao Chen B.D.  Qiaoxian Lin B.D.  Xiaoqiang Zheng B.D. |
| Investigational Product Name: | Sintilimab (Tyvyt®), Rituximab, Methotrexate, Temozolomide |
| Indication: | Newly Diagnosed Primary Central Nervous System Diffuse Large B-cell Lymphoma |
| Original Protocol: | 15-Aug-19 |
| Approval Date: | 12-Nov-19 |
| Funding Sponsor: | WU JIEPING MEDICAL FOUNDATION |
| Study sponsor and monitor: | The First Affiliated Hospital of Fujian Medical University |

# 1 SYNOPSIS

This is a single center phase II study to evaluate the efficacy and safety of sintilimab in combination with rituximab, high-dose methotrexate (HD-MTX) and temozolomide (RMT) in the first-line treatment in newly diagnosed primary central nervous system diffuse large B-cell lymphoma (PCNS-DLBCL), and to explore predictive biomarkers of response or resistance.

OVERVIEW OF STUDY DESIGN

In Part A of the study, newly diagnosed patients with PCNS-DLBCL will be enrolled. Patients will receive 6 cycles of sintilimab plus rituximab, HD-MTX and temozolomide (RMT) on 21-day cycles. At the beginning of the study, the first 6 subjects will be treated on the safety portion, and closely followed up and evaluated for the dose limiting toxicities (DLTs). If >1 DLTs are observed, dose adjustment will be considered before moving forward. If 1 or fewer patients experience DLT, continued enrollment onto the study will proceed according to a Simon’s Optimal two-stage design. In total, we aim to enroll 27 patients onto this part of the study. Investigators evaluated the efficacy according to response criteria of the International PCNS Lymphoma Collaborative Group (IPCG). Subjects who did not achieve partial remission after 4 cycles or did not achieve complete remission after 6 cycles or progress during treatment will withdraw from the study.

In Part B of the expansion study, a sample size of 20 patients was required to reject the null hypothesis of a 2-year progression-free survival (PFS) of 25% under the alternative hypothesis that the true 2-year PFS was 50%, with a two-sided 5% significance level and 80% power. Considering dropouts and withdrawals, the total sample size of this study is about 50 subjects.

SUBJECT POPULATION

INCLUSION CRITERIA

①Histologically confirmed diffuse large B-cell lymphoma limited to the central nervous system or eyes；

②Aged from 18 to 70 years；

③Eastern Cancer Cooperation Group (ECOG) performance status score 0-2;

④Estimated time of survival more than 12 weeks;

⑤Patient has at least one measurable lesion based on MRI or positive CSF cytology;

⑥No chemotherapy contraindications: neutrophil absolute count >= 1.5 x 10^9^ / L, platelet >= 100 x 10^9^ / L, ALT, AST <= 2 times of the upper limit of normal, serum total bilirubin <= 1.5 times of the upper limit of normal, creatinine clearance >= 50 ml / min;

⑦Adequate cardiopulmonary function;

⑧Pregnancy test of blood or urine is negative for women with reproductive potential. All patients agree to use adequate methods of contraception during the treatment period and until >= 12 months after the last chemotherapy；

⑨Ability and willingness to comply with the study protocol.

EXCLUSION CRITERIA

①Patients who have received other checkpoint inhibitors, such as PD-1 monoclonal antibody, PD-L1 monoclonal antibody and CTLA4 monoclonal antibody;

②Patients who have received radiotherapy or chemotherapy for lymphoma;

③Tumors involve other parts of the body other than the central nervous system;

④Previous medical history of other malignant tumors;

⑤Females of childbearing age with recent family planning;

⑥Active infection;

⑦Suspected human immunodeficiency virus (HIV)/AIDS infection;

⑧Uncontrolled or severe cardiovascular diseases;

⑨Possible allergy, allergy or intolerance to research drugs;

⑩Patients with interstitial lung disease;

⑪Severe diseases that may interfere with research;

⑫Patients received another research drug at the same time;

⑬According to the judgement of the researcher, there are other unsuitable situations for the patients to be enrolled in the group.

DOSAGE AND ADMINISTRATION

Subjects of newly diagnosed PCNSL were treated with sintilimab plus RMT regimen: sintilimab (200 mg) was intravenously administered on Day 0; rituximab (375 mg/m^2^) was intravenously administered on Day 0; methotrexate (3.0 g/m^2^) was intravenously administered for 4 hours on Day 1, and the dose for subjects ≥ 65 years old was adjusted to 1.0 g/m^2^ (the first 0.5 g/m^2^ finished in 0.5 hour, and the remaining in the next 3.5 hours). Each dose of MTX was followed 24 hours later by leucovorin 30 mg/m^2^. Leucovorin rescue was carried out every 6 hours until the MTX blood concentration was less than 0.1 μmol/L; temozolamide (150 mg/m^2^/d) was orally administered on Days 1-5. The sintilimab plus RMT regimen was given as six 21-days cycles. Intrathecal chemotherapy was not administered during treatment.

EVALUATIONS

The treatment response of patients receiving treatment was assessed according to the IPCG response criteria. The safety was measured by adverse events, laboratory test results, vital signs measurement, physical examination results and ECOG performance status level evaluation. Biopsy tissue samples, cerebrospinal fluid, blood and serum samples were used to explore the biomarkers of the response of subjects receiving sintilimab combined with RMT treatment.

# 2 BACKGROUND AND RATIONALE

# 2.1 Overview of Disease

Primary central nervous system lymphoma (PCNSL) is a rare type of extranodal non-Hodgkin lymphoma (NHL), which is usually confined to the brain, spinal cord, eyes without evidence of systemic involvement, accounting for 1% of all NHL and 4% of primary intracranial tumors [1–3]. PCNSL is associated with congenital or acquired immunodeficiency, especially human immunodeficiency virus (HIV)[4,5]. However, in recent years, the incidence rate of PCNSL has also increased in general population, with an annual incidence rate of 47/100,000 people, and the incidence rate of people over 60 years old has significantly increased [6,7]。

One of the typical histological characteristics of PCNSL is that the tumor cells are diffusely infiltrating, and typical vascular center infiltration can be seen at the edge. The tumor cells are often distributed around the blood vessels and arranged in a “sleeve like” structure, which can destroy the blood-brain barrier. Immunohistochemically, 95% of the pathological types were diffuse large B-cell lymphoma (DLBCL). Almost all PCNSL express pan-B cell markers, such as CD19, CD20, CD22 and CD79a, 50% - 80% of tumors express BCL6, more than 95% of tumors are MUM1 positive, and the positive rate of CD10 is only 10% - 20%. Therefore, PCNSL cells seem more likely to originate from activated B-cell like (ABC) [8]. The analysis of gene expression profile suggests that PCNSL is closer to memory B cells, and also supports that most PCNSL comes from late germinal centers or activated B cells[9,10]. PCNSL responds better to chemotherapy than other brain tumors but worse than systemic DLBCL [11,12]. The disease is highly invasive and its prognosis is poor. The median survival time is only 1.3 years, and the 5-year survival rate is 30.5% [7]. In addition, after receiving first-line treatment, 16.5% of PCNSL patients relapse, 29.0% of patients are refractory. The prognosis of refractory patients is very poor, with a median overall survival (OS) of only 2.1 months [13]. In the past few years, the induction chemotherapy of PCNSL has made important progress. However, compared with systemic DLBCL, complete remission (CR) rate is still low. Therefore, it is an important goal to improve the induction treatment of PCNSL to fill this blank area.

# 2.2 Current “standard” treatment

Despite a certain understanding of PCNSL’s pathogenesis, there is no standard first-line treatment. Because PCNSL is located deep within the skull and multiple lesions exist in some patients, surgical resection is risky and has many complications which is not conducive to long-term survival. Therefore, at present, biopsy of brain lesion with the least invasive approach is generally recommended to obtain a clear pathological diagnosis [1]. Whole brain radiotherapy (WBRT) was once the standard treatment for PCNSL. Although the rate of CR was as high as 90%, almost all patients recurred after receiving WBRT alone for several months. The median survival period of patients was only 12 months, and the long-term survival rate was 10%-29%. Moreover, the neurotoxicity caused by radiotherapy could significantly reduce the quality of life of patients [1,14,15]. Therefore, currently WBRT is not recommended as the routine treatment of PCNSL and just reserves for those who cannot tolerate systemic chemotherapy. Many treatments are currently used for PCNSL, including chemotherapy, targeted therapy, their combination with radiotherapy and autologous hematopoietic stem cell transplantation. However, there is no consensus on these treatments except for the importance of “high-dose systemic methotrexate”. The NCCN guidelines recognize that PCNSL is a rare disease, and it is difficult to conduct a strong randomized study. They believe that participation in clinical trials is encouraged as long as it is available and appropriate [16].

# 2.3 Methotrexate

Due to the existence of blood-brain barrier, many anti-tumor drugs cannot penetrate into the CNS and exert their anti-lymphoma effect. Methotrexate (MTX) is a cell cycle-specific anti-tumor drug that can inhibit cell growth by interfering with the synthesis of tetrahydrofolate. This small molecule drug crosses the blood-brain barrier relatively easily, although its drug concentration in cerebrospinal fluid is still less than 10% of the blood. Given that the adverse reaction of MTX is relatively mild, a larger dose can be used to increase the drug concentration in the brain and kill tumor cells.

High-dose methotrexate (HD-MTX) is extensively used for the treatment of PCNSL. The usual dosage of MTX is 1.0~8.0 g/m^2^. The evidence that beyond this dose can improve the efficacy is not sufficient at present. In the 1970s, HD-MTX monotherapy was considered to be an effective treatment to treat PCNSL. In the initial NABTT 96-07 study, methotrexate monotherapy was used as the standard treatment for PCNSL[17]. After 12 months of continuous methotrexate treatment without consolidation, the overall response rate (ORR) was 74%, the median progression-free survival (PFS) was 12.8 months, and the median OS was not reached at 22.8 months. In another study NOA-03 where 37 patients received single-drug MTX induction chemotherapy[18], Among these 11 patients with CR, 4 recurred and the time to recurrence were 7, 9 and 14 months, respectively for the three patients. The above studies showed that the efficacy of MTX monotherapy was limited and the recurrence rate is high in the short term. To further improve the survival of patients, other drugs that can penetrate the blood-brain barrier need to be added.

Clinicians have been trying to improve these results by adding other drugs or combining various consolidation therapies into HD-MTX induction. Further studies have also confirmed that when other chemotherapy drugs are added into HD-MTX, tumor response and survival results were significantly improved [19–22]. Therefore, currently relevant clinical treatment guidelines recommend HD-MTX based combination therapy as the cornerstone of PCNSL treatment. In addition, it should be noted that the dosage of MTX is related not only to the therapeutic effect but also to the side effects. HD-MTX may lead to serious adverse events such as renal failure, liver damage, digestive tract ulcer erosion, etc. The tolerance of HD-MTX in elderly patients is particularly noteworthy[23]. Therefore, this treatment must be carried out in the center that has the conditions to monitor the blood concentration of MTX and has rich experience in drug use. In a word, the existing HD-MTX-based treatment is difficult to balance the efficacy and safety, and its benefits to patients’ survival need further improvement.

# 2.4 Temozolomide

Temozolamide (TMZ) is a new type of oral imidazolium tetrazine alkylating agent. It has completely oral absorption, high bioavailability, good safety profile and can pass through the blood-brain barrier. It has been regarded as the first-line chemotherapy drug for the treatment of brain glioma in the past decade. The anti-tumor effect of TMZ depends on its ability to alkylate/methylate DNA, which usually occurs at the N7 or O6 position of guanine residues. This modification can destroy DNA and induce cancer cell death. TMZ has been recommended for the treatment of various intracranial tumors including PCNSL. Omuro et al. [24] used MT (MTX, TMZ) combination induction chemotherapy for newly diagnosed PCNSL patients. Of the 20 patients, 11 patients (55%) obtained CR, and 9 patients (45%) progressed, showing its its good effect in elderly patients with PCNSL. Wang et al. [20] compared MT and MA (high-dose MTX combined with high-dose cytarabine) chemotherapy regimen in 41 patients with PCNSLand found that MT regimen has equivalent efficacy but superior tolerance to MT regimen.

# 2.5 Rituximab

Rituximab is an anti-CD20 monoclonal antibody, which has a significant effect in the treatment of DLBCL outside the central nervous system. However, due to its large molecular weight and low permeability of the blood-brain barrier, its clinical application is still controversial. Studies have shown that when administered intravenously at a dose of 375 mg/m^2^, the concentration of rituximab in cerebrospinal fluid is 0.1% of the plasma concentration [25], but some researchers believe that rituximab can reach the therapeutic concentration at the CNS tumor site after the blood-brain barrier is broken [26]. In Batchelor et al study, 12 patients with recurrent PCNSL were treated with rituximab 375mg/m^2^ once a week, up to 8 times, with a remission rate of 36% and a median OS of 20.9 months [25]. Holdhoff et al. [27] reviewed 81 elderly patients, 54 of whom received HD-MTX and 27 received HD-MTX+R. The CR rate of the two groups was 36% vs 73% (P=0.0145), the median PFS was 4.5 months vs 26.7 months (P=0.003), and the OS was 16.3 months vs not reached (P=0.01), respectively, indicating that adding rituximab to HD-MTX could improve both CR rate and PFS, but not the OS.

The results of IELSG32 randomized trial showed that adding rituximab and thiotepa to the standard MA (MATRix protocol) increased ORR increased from 53% to 87%, the CR rate from 23% to 49%, the 2-year PFS rate from 36% to 61%, and the 2-year OS rate from 42% to 69% [22]. However, the toxicity and side effects of MATRix regimen were higher, especially the incidence of severe bone marrow suppression and febrile neutropenia. HOVON 105/ALLG NHL 24 was a randomized phase III trial comparing MBVP (MTX, carmustine, teniposide, prednisone) and R-MBVP in the treatment of 200 newly diagnosed PCNSL [28]. There was no statistical difference in the 1-year event-free survival (EFS) rate (49% vs 52%) and 3-year OS rate (61% vs 58%), indicating no significant benefit of adding rituximab to the chemotherapy in PCNSL patients. Although there is no conclusive evidence of the benefit of rituximab in PCNSL patients, most treatment centers have included it in the initial treatment of PCNSL.

# 2.6 PD-1 Inhibitors

Immune checkpoints are essential for efficient T-cell activation, but also for maintaining self-tolerance and protecting tissues from damage caused by the immune system, and for providing protective immunity. One of the most critical checkpoint pathways is mediated by programmed cell death ligand 1/2 (PD-L1/PD-L2). PD-1 is highly expressed in B cells, activated T cells, dendritic cells and NK cells, while tumor cells escape immune surveillance by overexpressing PD-L1[29]。

Improvement and application of genome sequencing has provided in-depth insights into the pathogenesis of PCSNL. PCNSL has different genetic characteristics from DLBCL elsewhere. Its genome is more unstable and more often accompanied by copy number changes or translocation of 9p24.1/PD-L1/PD-L2, which leads to up-regulation of PD-L1 expression. This up-regulation may be the genetic basis of immune escape [12]. It has been found that the expression rate of PD-1 in tumor infiltrating lymphocytes (TIL) and PD-L1 in tumor cells is higher than that of systemic DLBCL (58% and 37% respectively) [30]. The expression of PD1 in TIL was associated with the expression of PDL1 in tumor cells (P=0.001), and the presence of PD-1 positive TIL was associated with poor overall survival rate (P=0.011) [30]. Cho et al. [31] retrospectively analyzed 76 newly diagnosed PCNSL patients who received high-dose methotrexate-based chemotherapy. The expression rates of PD-1, PD-L1 and PD-L2 were 7.9%, 13.2% and 42.1%, respectively. In multivariate analysis, high PD-1 (P=0.007) and Memory Sloan Kettering Cancer Center (MSKCC) score (P=0.019) were independently associated with poor OS, and high PD-1 expression was again with poor PFS of PCNSL patients (P=0.028). Therefore, PD1/PDL1 pathway plays an important role in PCNS-DLBCL, providing a new target of treatment for PCNSL.

With the advent of PD-1 inhibitors, immunotherapy has become a new hope for many cancer patients. Clinically, blocking PD-1 or PD-L1 with monoclonal antibodies has significant clinical activity in patients with metastatic melanoma, renal cell carcinoma, non-small cell lung cancer, bladder cancer, head and neck cancer (HNSCC), Hodgkin lymphoma and other tumors. In the preclinical model of CNSL with normal immune function, 50% of the mice obtained imaging complete remission after receiving PD-1 monoclonal antibody treatmentwhich was confirmed in biopsy immunohistochemistry [32], which laid a theoretical foundation for the clinical trial of PD-1 inhibitor treating PCNSL. A retrospective study by Nayak et al. [33] reported that five patients with recurrent/refractory PCNSL and primary testicular lymphoma (PTL) received intravenous injection of nivolumab. All five patients had clinical and imaging reactions to PD-1 inhibitors, and three patients remained progression-free at 13+ to 17+ months. The results showed that nivolumab played an active role in recurrent/refractory PCNSL and PTL, and supported further research on the PD-1 blockade in these diseases. Terziev et al. [34] reported a PCNSL patient with multiple relapses who received ASCT treatment. After achieving CR, he received 12 months of nivolumab maintenance treatment and obtained long-term CR.

Developed jointly by Lilly Pharmaceutical and Innovent Biologics, sintilimab is a humanized IgG4 monoclonal antibody PD-1 inhibitor and was approved for marketing in December 2018. It has high specificity in binding with PD-1 receptor, thus blocking its interaction with PD-L1 and PD-L2. The National Drug Administration (NMPA) has approved its use in the treatment of recurrent refractory classic Hodgkin lymphoma and other for various solid tumors, including non-small cell lung cancer and esophageal cancer. FDA of the United States accepted sintilimab's application for a new research drug in January 2018 [35]. Therefore, the efficacy and safety of sintilimab in PCNSL deserve further exploration.

# 2.7 Study Rationale

The therapeutic effect of rituximab combined with MT (HD-MTX and temozolomide) on PCNSL has attracted much attention. In 2012, Wieduwilt et al. [36] proposed a RMT protocol (rituximab combined with HD-MTX 8.0g/m^2^ and temozolomide) as an induction protocol to treat PCNSL. After receiving RMT treatment, the ORR was 58% and the CR rate 52%. Regardless of whether a combination of high-dose etoposide and cytarabine (EA) was used for consolidation therapy, the 2-year PFS and OS rates were 45% and 58%, respectively. This treatment scheme rarely caused bone marrow suppression and other serious adverse events. In the CALGB 50202 trial in 2013 [21], 44 newly diagnosed PCNSL patients received RMT regimen (rituximab, HD-MTX 8.0g/m^2^, and 150 g/m^2^ temozolomide for five consecutive days) induction treatment followed by EA consolidation, with ORR 77%, CR rate 66%, and 2-year PFS rate 57%. In the clinical study of NRG Oncology RTOG 0227 [37], PCNSL patients received induction chemotherapy of RMT regimen (rituximab, 3.5 g/m^2^ HD-MTX at the first, third, fifth, seventh and ninth weeks, and 200 g/m^2^/d temozolomide for five consecutive days) followed by whole-brain radiotherapy. The ORR of 35 patients who completed RMT chemotherapy was 85%, the CR rate 51%, and the 2-year OS rate 80.8%.

Special attention should be given to older patients who are vulnerable to intensive chemotherapy. In a phase II study of PCNSL patients over 60 years old carried out by the European Cancer brain tumor Research and Treatment Organization, patients received HD-MTX (1.0g/m^2^), lomustine, procarbazine, methylprednisolone, and intrathecal chemotherapy with MTX and cytarabine [38]. The ORR rate of patients was 48%, the CR rate was 42%. Overall median survival time was 14.3 months, and 1-year PFS was 40%. Except for a fatal pulmonary embolism, no toxic death was reported during chemotherapy. Therefore, HD-MTX (1.0 g/m^2^) based combination may be effective and safe for elderly PCNSL patients.

In the mouse model of glioma in situ, temozolomide can up-regulate the expression of PD-L1 in glioma cells, leading to immune escape [39]. Compared with other groups (control group, single drug temozolomide, single drug PD-1 antibody), the combined treatment group (temozolomide combined with PD-1 antibody) significantly improved the OS rate (P<0.01), significantly reduced tumor volume or size (P<0.01), and significantly increased the number of CD4 and CD8 infiltrating cells in brain tumors (P<0.01)[40]. These provided a theoretical basis for the combination of temozolomide and PD-1 antibody in the treatment of tumor.

The previous study found that the deletion of B cells did not hinder the anti-tumor activity of PD-1 inhibitor [41]. PD-1 inhibitors have been studied in patients treated with B-cell consumption antibodies, such as primary mediastinal large B-cell lymphoma previously treated with rituximab [42]. A phase II clinical study [43] of PD-1 antibody combined with rituximab in the treatment of follicular lymphoma showed that , among the follicular lymphoma patients who had received 1-4 cycles of rituximab in the past, 29 were eligible for evaluation. The ORR was 66%, the CR 52%, and 86% with tumor regression, suggesting that the combination of PD-1 antibody and rituximab might have synergistic anti-tumor effect. Many ongoing clinical trials are evaluating the activity of PD-1 inhibitor combined with rituximab in the treatment of B-cell lymphoma (such as NCT03401853 and NCT02446457).

In addition, a retrospective analysis found that high PD-1 expression is an important adverse prognostic factor in PCNSL patients receiving HD-MTX treatment [31]. This raised a question of whether adding PD-1 antibody to HD-MTX based treatment can overcome the adverse prognosis caused by high PD-1 expression. Another retrospective study by Nayak et al. [33] reported that nivolumab monotherapy had achieved good results in the treatment of 5 cases of relapsed refractory PCNSL/PTL. At present, however, study of PD-1 inhibitor combined with chemotherapy in newly diagnosed PCNSL is rare. We hypothesized that, by improving immune surveillance, the combination of sintilimab and rituximab, methotrexate and temozolomide will help to exert the anti-PCNSL activity. In this phase II study, we aimed to evaluate the efficacy and safety of the first-line treatment of PCNSL with sintilimab plus rituximab, HD-MTX and temozolomide (RMT), and to explore potential biomarkers.

# 3 OBJECTIVES AND ENDPOINTS

# 3.1 Primary Objective

Evaluate the overall response rate to sintilimab plus RMT in the treatment of newly diagnosed PCNSL

# 3.2 Secondary Objectives

- Evaluate the duration of response to sintilimab plus RMT in the treatment of newly diagnosed PCNSL
- Evaluate PFS to sintilimab plus RMT in the treatment of newly diagnosed PCNSL
- Evaluate OS to sintilimab plus RMT in the treatment of newly diagnosed PCNSL
- Evaluate safety to sintilimab plus RMT in the treatment of newly diagnosed PCNSL

# 3.3 Exploratory Objectives

We propose a series of studies to evaluate the association or correlation of the following biomarkers with ORR and PFS:

- The mutational pattern detected by the next generation sequencing (whole-exome sequencing)
- Tumor-specific cell-free DNA of cerebrospinal fluid and peripheral blood at baseline, after 4 cycles, 6 cycles, and three months after end of treatment
- Immune repertoire of peripheral blood at baseline, after 4 cycles, 6 cycles, and three months after end of treatment
- Immunohistochemistry of biopsy tissue (including but not limited to routine detection, PD-1, PD-L1, tumor infiltrating lymphocyte related immunophenotype, etc.)
- Lymphocyte subsets of peripheral blood at baseline, after 1-6 cycles of sintilimab plus RMT
- Cytokine of cerebrospinal fluid and peripheral blood at baseline, after 4 cycles, 6 cycles, and three months after end of treatment
- Proteomic analysis of cerebrospinal fluid and peripheral blood at baseline, after 4 cycles, 6 cycles, and three months after end of treatment
- Soluble PD-L1 level in peripheral blood at baseline, after 4 cycles, 6 cycles, and three months after end of treatment
- Quantitative FDG PET metrics, including metabolic tumor volume (MTV), standard uptake value (SUV), and total lesion glycolysis (TLG), at baseline, after 4 cycles, and following 6 cycles of treatment
- Quantitative including but not limited to gadolinium-enhanced MRI metrics, diffusion weighted imaging (DWI), perfusion weighted imaging (PWI) and intravoxel incoherent motion imaging (IVIM) at baseline, after 1-6 cycles of treatment
- Quality of life (QOL) at baseline and during treatment
- Mini-Mental State Examination (MMSE) at baseline and during treatment

# 4 OVERVIEW OF STUDY DESIGN/INTERVENTION

# 4.1 Study Design

This is a single-center, investigator-initiated, Phase II study of adult subjects who were newly diagnosed PCNSL treated with sintilimab plus rituximab, HD-MTX and temozolomide (RMT). In Part A of this study, patients with newly diagnosed PCNSL will be enrolled and will receive 6 cycles of sintilimab plus RMT on 21-day cycles. Initially, 6 patients will be treated on the safety portion of the study and evaluated for dose-limiting toxicities (DLTs). If 1 or fewer patients experience dose-limiting toxicity (DLT), the treatment will be considered tolerable, and enrollment onto the study will proceed according to Simon’s Optimal two-stage design. If >1 DLTs are observed, dose adjustment will be considered before continuing the study. The trial was designed to have 80% power and a one-side type I error rate of 5% to reject the null hypothesis of a overall response rate (ORR) of 58% when the true ORR is 83%. In total, we aim to enroll 27 subjects onto the Part A. Response definition was based on changes in tumor size of enhanced lesions on gadolinium-enhanced MRI, ocular examination and CSF exams, following the International PCNS Lymphoma Cooperation Group (IPCG) response criteria. Subjects who did not reach partial remission (PR) after 4 cycles of treatment, or did not reach complete remission (CR) after 6 cycles of treatment, or had progress during the treatment, will be withdrew and treated according to Section 8. In the Part B of the study, an additional 20 patients will be enrolled onto an expansion cohort and receive sintilimab plus RMT regimen. Considering dropouts and withdrawals, the total sample size of this study is about 50 subjects.

# 4.2 Intervention

# 4.2.1 Dosing and Schedule of Treatment

Subjects of newly diagnosed PCNSL will be treated with sintilimab plus RMT regimen: sintilimab (200 mg) was intravenously administered on Day 0; rituximab (375 mg/m^2^) was intravenously administered on Day 0; methotrexate (3.0 g/m^2^) was an intravenously administered for 4 hours on Day 1, and the dose for subjects ≥ 65 years old was adjusted to 1.0 g/m^2^ (the first 0.5 g/m^2^ finished in 0.5 hour, and the remaining in the next 3.5 hours). Each dose of MTX was followed 24 hours later by leucovorin 30 mg/m^2^. Leucovorin rescue was carried out every 6 hours until the MTX blood concentration was less than 0.1 μmol/L; temozolamide (150 mg/m^2^/d) was orally administered on Days 1-5. The sintilimab plus RMT regimen will be given as six 21-days cycles. Intrathecal chemotherapy was not administered during treatment.

The administration of infusion time and infusion related reactions of methotrexate and rituximab should follow the guidelines of the organization. For the infusion reaction related to sintilimab, please refer to dose adjustment guidelines for drug-related adverse events in Section 10.3.

# 4.2.2 Safety Window and DLT definition

All toxicities will be graded according to the National Cancer Institute Common Terminology Criteria for Adverse Events (NCI CTCAE), Version 4.03 (Appendix 2). Initially, 6 patients with PCNSL will be treated on the safety run-in cohort of Part A and observed for DLTs. The DLT evaluation period is from Day 1 to Day 21 of the first cycle. A DLT is defined as any Grade 3 or greater AE during the 3-week DLT observation period which is judged to be related to any to study drug (sintilimab, rituximab, HD-MTX and temozolomide). If any enrolled patient in safety run-in cohort of Part A withdraws from the study during the DLT period, this patient will be replaced unless discontinuation is for DLT. If 1 or fewer patients experience dose limiting toxicity (DLT), enrollment onto the study will proceed according to a phase II, Simon’s two-stage design.

The following are exceptions to the DLT definition and are NOT considered a DLT:

- Grade 3 anemia. However, Grade 3 hemolytic anemia that is medically significant, requires hospitalization or prolongation of existing hospitalization, is disabling, or limits self-care activities of daily life (ADLs) is considered a DLT.
- Grade 3 indirect/unconjugated hyperbilirubinemia that resolves to ≤ Grade 2 with supportive care within 1 week and is not associated with other clinically significant consequences.
- Isolated Grade 3 electrolyte abnormalities that resolve to ≤ Grade 2 with supportive care within 1 week and are not associated with other clinically significant consequences.
- Grade 3 elevation in alanine aminotransferase, aspartate aminotransferase, or alkaline phosphatase that resolves to ≤ Grade 2 with supportive care within 1 week and is not associated with other clinically significant consequences.
- Grade 3 nausea, vomiting, or diarrhea that resolves to ≤ Grade 2 with supportive care within 72 hours.
- Grade 3 fatigue that resolves to ≤ Grade 2 within 2 weeks on study.

• Grade 3 sintilimab infusion reactions in the absence of pretreatment.

• Grade 3 infusion reactions attributed to rituximab; when rituximab and sintilimab are both dosed, an infusion reaction can only be attributed to rituximab alone if the infusion reaction occurs after the start of rituximab infusion.

# 4.2.3 Safety Monitoring Beyond After Completion of Safety Window

During the study, patients will continue to be monitored for DLTs, adverse events, and events of interest throughout treatment with sintilimab and RMT until 100 days following the last dose of sintilimab. Total planned enrollment is 50 patients and events of interest occurring in up to 15% of the 50 patients enrolled will warrant protocol modification or study closure. We plan to assess the need for protocol modification well before 15% of patients experience events of interest. Therefore, the following events of interest (throughout the treatment with sintilimab and RMT until 100 days following the last dose of sintilimab) will lead to a pause in enrollment and safety analysis and consideration for protocol modification or closure: 3 or more DLTs among patients enrolled after the safety window, 3 or more events of grade ≥3 pneumonitis or grade ≥4 hepatotoxicity, and any non-relapse related death.

# 5 SELECTION, WITHDRAWAL, AND REPLACEMENT OF SUBJECTS

# 5.1 SUBJECT POPULATION

# 5.1.1 Inclusion Criteria

Each potential subject must satisfy all of the following criteria to be enrolled in the study:

①Histologically confirmed diffuse large B-cell lymphoma limited to the central nervous system or eyes；

②Aged from 18 to 70 years；

③Eastern Cancer Cooperation Group (ECOG) performance status score 0-2;

④Estimated time of survival more than 12 weeks;

⑤At least one measurable lesion based on MRI or positive CSF cytology;

⑥No chemotherapy contraindications: neutrophil absolute count >= 1.5 x 10^9^ / L, platelet >= 100 x 10^9^ / L, ALT and AST <= 2 times of the upper limit of normal, serum total bilirubin <= 1.5 times of the upper limit of normal, creatinine clearance >= 50 ml / min;

⑦Adequate cardiopulmonary function;

⑧Pregnancy test of blood or urine is negative for women of reproductive potential. All patients agree to use adequate methods of contraception during the treatment period and until >= 12 months after the last chemotherapy；

⑨Ability and willingness to comply with the study protocol.

# 5.1.2 Exclusion criteria

Any potential subject who meets any of the following criteria will be excluded from participating in the study:

①Patients who have received other checkpoint inhibitors, such as PD-1 monoclonal antibody, PD-L1 monoclonal antibody and CTLA4 monoclonal antibody;

②Patients who have received radiotherapy or chemotherapy for lymphoma;

③Tumors involve other parts of the body other than the central nervous system;

④Previous medical history of other malignant tumors;

⑤Females of childbearing age with recent family planning;

⑥With active infection;

⑦Suspected human immunodeficiency virus (HIV)/AIDS infection;

⑧Uncontrolled or severe cardiovascular diseases;

⑨Possible allergy, allergy or intolerance to research drugs;

⑩Patients with interstitial lung disease;

⑪Severe diseases that may interfere with research;

⑫Patients received another research drug at the same time;

⑬According to the judgement of the researcher, there are other unsuitable situations for the patients to be enrolled in the study.

# 5.2 Premature Withdrawal from the Study or from Study Treatment

# 5.2.1 Reasons for Premature Withdrawal or Discontinuation of Study Treatment

A patient has the right to voluntarily discontinue study treatment or withdraw from the study at any time, for any reason, and without repercussion.

The investigator and sponsor have the right to discontinue a patient from study treatment or withdraw a patient from the study at any time.

Reasons for discontinuation of study treatment or withdrawal from the study may include, but are not limited to:

• Patient withdrawal of consent at any time

• Any medical condition that the investigator or sponsor determines may jeopardize the patient’s safety if he or she continues in the study or continues treatment with study drug

• The investigator or sponsor determines it is in the best interest of the patient

• Patient noncompliance (eg, not complying with protocol required visits, assessments, and dosing instructions)

• Pregnancy

# 5.2.2 Withdrawal from Study Participation

During the treatment period and follow-up period, a patient who withdraws consent to continue participation in the study will not be followed for any reason after consent has been withdrawn. Every effort should be made to obtain information on patients who withdraw from the study.

An excessive rate of withdrawals would render the study uninterpretable; therefore, unnecessary withdrawal of patients should be avoided.

# 5.2.3 Discontinuation of Study Treatment

If the subject has to terminate the treatment before the end of the treatment, it will not be considered as automatic withdrawal from the study. If the subject must terminate the study treatment before the end of the treatment plan, the termination of the treatment will not cause the subject to automatically withdraw from the study. After termination of treatment, the subjects will enter the follow-up period. The end of treatment visit and follow-up visit evaluation should be continued according to the time and event schedule. If the study treatment is stopped due to reasons other than the progress of the disease, the disease assessment will continue according to the time and event schedule.

In case of any of the following conditions, the subject must stop the study treatment:

- The investigator assumes that for safety or tolerance reasons (e.g., AE), it is most beneficial for the subject to stop the study and treatment.
- Subjects withdraw the informed consent to participate in the study
- Subjects receive treatment with banned drugs
- Subjects receive other treatment not specified in the study protocol
- Subjects have intolerable toxicity
- Disease progression of subjects

The main reason for terminating the treatment should be recorded in the CRF. The investigator will record the disease progress (such as filling in the disease progress form) as soon as possible. If the subject terminates the treatment at any time for reasons other than disease progression, the investigator should also record the end of treatment as soon as possible within 48 hours.

The investigator will determine whether the study treatment should be terminated, and the subjects will be followed up after the termination of the treatment.

# 5.3 Replacement of Patients

Before the completion of the DLT observation period (Day 1 to Day 21 of the first cycle), any subjects who stopped treatment or withdrew from the study for any reason other than DLT defined in the protocol or other AEs leading to the suspension of the treatment will be replaced. Each replacement subject will be assigned a unique subject number and will be treated at the same dose level as the replacement early withdrawal subject. Subjects who stopped treatment after the DLT observation period will not be replaced.

# 6 THERAPEUTIC/DIAGNOSTIC AGENTS

# 6.1 Sintilimab

Sintilimab is a full-human IgG4 monoclonal antibody. Sintilimab can bind to PD-1, block the interaction between PD-1 and its ligand, and help restore the anti-tumor response of T cells. Sintilimab injection is an innovative biological drug jointly developed by Lilly Pharmaceutical and Innovent Biologics in China. On December 24, 2018, sintilimab was officially listed with the approval of NMPA.

# 6.1.1 General Information

Generic Name: Sintilimab injection

Commercial Name: Tyvyt® Solution for Infusion, 10ml:100mg vial

# 6.1.2 Formulation

Sintilimab is a sterile, preservative-free, clear to slightly opalescent, colorless to slightly yellow solution that requires dilution for intravenous infusion. Each vial contains 100 mg of sintilimab in 10 ml of solution. Every 1 ml of solution contains 10 mg of sintilimab, and the ingredients are as follows: mannitol, histidine, sodium citrate (dihydrate), sodium chloride, disodium edetate, polysorbate 80, citric acid (monohydrate), and water for injection.

# 6.1.3 Clinical Pharmacology

PD-1 (programmed death receptor 1), also known as CD279, is a co-inhibitory receptor expressed on the surface of T cells, B cells, monocytes and natural killer cells. It has two binding ligands - Programmed Death Ligand 1 (PD-L1) and PD-L2 (B7 family) expressed on normal cells. The combination of PD-1 with any ligand will inhibit T cell activity, induce T cell tolerance, inhibit proliferation, reduce T cell immune response and induce cell death, thus preventing the killing of normal cells activated by immune cells. The PD-1 ligand of some tumor cells is up-regulated, which can inhibit the immune monitoring of tumor by activated T cells through signal transduction.

Sintilimab is a kind of monoclonal antibody of fully humanized immunoglobulin G4 (IgG4), which can specifically bind PD-1 molecule on the surface of T cells, thus blocking the PD-1/PD-L1 pathway leading to tumor immune tolerance, reactivating the anti-tumor activity of lymphocytes, and thus achieving the purpose of treating tumor.

# 6.1.4 Pharmacokinetics and Drug Metabolism

**Absorption**: Sintilimab is administered by intravenous infusion. The serum concentration gradually rises from the beginning of infusion, reaches the peak after the end of infusion, and then decreases slowly.

**Distribution**: The distribution volume (Vss) of sintilimab in stable state is small (about 4.7L; coefficient of variation (CV): 22%). As expected by the antibody, Sintilimab will not bind to plasma protein in a specific way.

**Metabolism**: Sintilimab is catabolized through non-specific pathways. Metabolism does not contribute to its clearance.

**Clearance**: The mean baseline clearance rate (coefficient of variation) of sintilimab was 0.32 days (59%); The mean steady-state clearance rate (coefficient of variation) was 0.21 days (72%), and the mean steady-state end-elimination half-life (coefficient of variation) was 20.9 days (46%).

**Drug interaction**: Because the clearance of monoclonal antibodies does not involve cytochrome P450 (CYP450)-mediated metabolism, the inhibition or induction of these enzymes by the combined drugs is not expected to affect the pharmacokinetics of sintilimab.

# 6.1.5 Supplier

Innovent Biologics.

# 6.2 Rituximab

Refer to package insert.

# 6.3 Methotrexate

Refer to package insert.

# 6.4 Temozolomide

Refer to package insert.

# 7 INTERVENTION ALLOCATION AND BLINDING

This is an open label study and will not use blind method. Randomization is not used in this study. If all inclusion/exclusion criteria are met, the subjects will receive the treatment of the study protocol.

# 8 EVALUATION PRIOR TO TREATMENT

- The patient needs to undergo biopsy for pathological diagnosis and confirm that the pathological type is diffuse large B-cell lymphoma. If biopsies have been performed, 10-20 unstained sections from archived tissues are required for relevant studies.
- ECOG performance status evaluation
- Vital signs, height, weight
- Gadolinium-enhanced magnetic resonance imaging
- PET/CT
- Bone marrow examination
- Cerebrospinal fluid evaluation when the condition allows and the patient agrees
- Ophthalmic examination
- Complete blood count
- Routine biochemistry of serum
- Hepatitis B surface antigen, core antibody, and hepatitis B DNA
- Hepatitis C antibody
- HIV antibody
- Pregnancy test - urine or serum (women of reproductive age)
- Quality of life assessment (QOL, according to EORTC QLQ-C30)
- Mini-mental state examiniation (MMSE)

# 9 TREATMENT/INTERVENTION PLAN

Subjects will receive a total of 6 cycles of treatment with sintilimab, rituximab, methotrexate and temozolomide. Gadolinium-enhanced brain MRI was performed less than 14 days before chemotherapy (immunotherapy), and the treatment response was evaluated by repeated MRI on the sixth day of the first cycle of chemotherapy (immunotherapy), before the second to sixth cycle of chemotherapy (immunotherapy), and 21 days after the final treatment protocol. Following the IPCG response criteria, the definition of treatment response is based on the tumor size change of the enhancement focus in MRI, eye examination and CSF examination. Subjects who obtained ≥ PR in the mid-term evaluation after 4 cycles of treatment will continue to receive the original treatment, and those who did not reach PR will withdraw from the treatment. After 6 cycles of treatment, CR or CRu subjects were evaluated at the end of the treatment period and entered the follow-up period, and those who do not reach CR or CRu will receive salvage treatment.

# 10 EVALUATION DURING TREATMENT/INTERVENTION

**Table 1. Evaluations During Treatment/Intervention**

|  | Screening period | Treatment period ^a^ (21-day Cycles) | | | | | | | | | | | | | | | | | | | | | | | | End of treatment | Follow -up period |
| --- | --- | --- | --- | --- | --- | --- | --- | --- | --- | --- | --- | --- | --- | --- | --- | --- | --- | --- | --- | --- | --- | --- | --- | --- | --- | --- | --- |
|  |  | Cycle 1 | | | | Cycle 2 | | | | Cycle 3 | | | | Cycle 4 | | | | Cycle 5 | | | | Cycle 6 | | | |  |  |
| Day | -28 | 1 | 1 | 2 | 2-6 | 1 | 1 | 2 | 2-6 | 1 | 1 | 2 | 2-6 | 1 | 1 | 2 | 2-6 | 1 | 1 | 2 | 2-6 | 1 | 1 | 2 | 2-6 | 21 days (+7) post last protocol therapy dose | Every 3 months up to 2 years |
| Test/Evaluation/Treatment |  |  |  |  |  |  |  |  |  |  |  |  |  |  |  |  |  |  |  |  |  |  |  |  |  |  |  |
| Informed Consent | X |  |  |  |  |  |  |  |  |  |  |  |  |  |  |  |  |  |  |  |  |  |  |  |  |  |  |
| History and Physical examination | X | X |  |  |  | X |  |  |  | X |  |  |  | X |  |  |  | X |  |  |  | X |  |  |  | X | X |
| Vital signs ^b^ | X | X | X | X | X | X | X | X | X | X | X | X | X | X | X | X | X | X | X | X | X | X | X | X | X | X | X |
| Record Concomitant Medications | X |  |  |  |  | X |  |  |  | X |  |  |  | X |  |  |  | X |  |  |  | X |  |  |  |  |  |
| Urine or Serum β-HCG ^c^ | X |  |  |  |  |  |  |  |  |  |  |  |  |  |  |  |  |  |  |  |  |  |  |  |  |  |  |
| ECOG Performance Status | X |  |  |  |  | X |  |  |  | X |  |  |  | X |  |  |  | X |  |  |  | X |  |  |  | X |  |
| KPS | X |  |  |  |  | X |  |  |  | X |  |  |  | X |  |  |  | X |  |  |  | X |  |  |  | X |  |
| IELSG Risk Score | X |  |  |  |  |  |  |  |  |  |  |  |  |  |  |  |  |  |  |  |  |  |  |  |  |  |  |
| CBC | X | X | X | X | X | X | X | X | X | X | X | X | X | X | X | X | X | X | X | X | X | X | X | X | X |  |  |
| LDH | X | X | X | X | X | X | X | X | X | X | X | X | X | X | X | X | X | X | X | X | X | X | X | X | X |  |  |
| Serum Chemistry | X | X | X | X | X | X | X | X | X | X | X | X | X | X | X | X | X | X | X | X | X | X | X | X | X |  |  |
| Thyroid Function (TSH, FT3, FT4) | X |  |  |  |  | X |  |  |  | X |  |  |  | X |  |  |  | X |  |  |  | X |  |  |  |  |  |
| ECG | X |  |  |  |  | X |  |  |  | X |  |  |  | X |  |  |  | X |  |  |  | X |  |  |  |  |  |
| Hepatitis B and C and HIV 1/2 antibodies | X |  |  |  |  |  |  |  |  |  |  |  |  |  |  |  |  |  |  |  |  |  |  |  |  |  |  |
| Ophthalmic Examination | X |  |  |  |  |  |  |  |  |  |  |  |  |  |  |  |  | X |  |  |  |  |  |  |  |  |  |
| Bone Marrow Smear / Flow Cytometry / Biopsy | X |  |  |  |  |  |  |  |  |  |  |  |  |  |  |  |  |  |  |  |  |  |  |  |  |  |  |
| Gadolinium-Enhanced Whole-Brain MRI | X ^d^ |  |  |  | X | X ^e^ |  |  |  | X ^e^ |  |  |  | X ^e^ |  |  |  | X ^e^ |  |  |  | X ^e^ |  |  |  | X |  |
| ^18F^FDG-PET/CT | X |  |  |  |  |  |  |  |  |  |  |  |  |  |  |  |  | X ^e^ |  |  |  |  |  |  |  |  |  |
| CT chest | X ^d^ |  |  |  |  | X ^e^ |  |  |  | X ^e^ |  |  |  | X ^e^ |  |  |  | X ^e^ |  |  |  | X ^e^ |  |  |  |  |  |
| Echocardiogram | X |  |  |  |  |  |  |  |  |  |  |  |  |  |  |  |  |  |  |  |  |  |  |  |  |  |  |
| EORTC QLQ-C30 | X |  |  |  |  | X |  |  |  | X |  |  |  | X |  |  |  | X |  |  |  | X |  |  |  | X |  |
| EORTC QLQ-BN20 | X |  |  |  |  | X |  |  |  | X |  |  |  | X |  |  |  | X |  |  |  | X |  |  |  | X |  |
| MMSE | X |  |  |  |  | X |  |  |  | X |  |  |  | X |  |  |  | X |  |  |  | X |  |  |  | X |  |
| DLT assessment ^f^ |  | X | X | X | X | X |  |  |  |  |  |  |  |  |  |  |  |  |  |  |  |  |  |  |  |  |  |
| Histopathology and Molecular Markers | X |  |  |  |  |  |  |  |  |  |  |  |  |  |  |  |  |  |  |  |  |  |  |  |  |  |  |
| Whole Exon Sequencing of Biopsy Tissue | X |  |  |  |  |  |  |  |  |  |  |  |  |  |  |  |  |  |  |  |  |  |  |  |  |  |  |
| Peripheral Blood Lymphocyte Subsets Analysis | X |  |  |  |  | X |  |  |  | X |  |  |  | X |  |  |  | X |  |  |  | X |  |  |  | X ^g^ | X ^g^ |
| Peripheral Blood Cytokine | X |  |  |  |  | X |  |  |  | X |  |  |  | X |  |  |  | X |  |  |  | X |  |  |  | X ^g^ | X ^g^ |
| Peripheral Blood ctDNA | X |  |  |  |  |  |  |  |  |  |  |  |  |  |  |  |  | X |  |  |  |  |  |  |  | X ^g^ | X ^g^ |
| Peripheral Blood PD-L1 Concentration | X |  |  |  |  |  |  |  |  |  |  |  |  |  |  |  |  | X |  |  |  |  |  |  |  | X ^g^ | X ^g^ |
| Peripheral Blood Immune Repertoire | X |  |  |  |  |  |  |  |  |  |  |  |  |  |  |  |  | X |  |  |  |  |  |  |  | X ^g^ | X ^g^ |
| CSF Routine / Cytology / Biochemistry / Flow Cytometry | X ^h^ |  |  |  |  |  |  |  |  |  |  |  |  |  |  |  |  | X ^h^ |  |  |  |  |  |  |  | X ^g,h^ | X ^g,h^ |
| CSF Cytokine | X ^h^ |  |  |  |  |  |  |  |  |  |  |  |  |  |  |  |  | X ^h^ |  |  |  |  |  |  |  | X ^g,h^ | X ^g,h^ |
| CSF ctDNA | X ^h^ |  |  |  |  |  |  |  |  |  |  |  |  |  |  |  |  | X ^h^ |  |  |  |  |  |  |  | X ^g,h^ | X ^g,h^ |
| Study Drug Administration |  |  |  |  |  |  |  |  |  |  |  |  |  |  |  |  |  |  |  |  |  |  |  |  |  |  |  |
| Sintilimab |  | X | X |  |  | X | X |  |  | X | X |  |  | X | X |  |  | X | X |  |  | X | X |  |  |  |  |
| Rituximab |  |  |  |  |  |  |  |  |  |  |  |  |  |  |  |  |  |  |  |  |  |  |  |  |  |  |  |
| Methotrexate |  |  |  | X |  |  |  | X |  |  |  | X |  |  |  | X |  |  |  | X |  |  |  | X |  |  |  |
| Temozolomide |  |  |  |  | X |  |  |  | X |  |  |  | X |  |  |  | X |  |  |  | X |  |  |  | X |  |  |
| Record Adverse Events |  | After signing the informed consent form until 28 days after the last administration. | | | | | | | | | | | | | | | | | | | | | | | | |  |
| Survival Status / Sequential Anti-lymphoma Therapy |  |  |  |  |  |  |  |  |  |  |  |  |  |  |  |  |  |  |  |  |  |  |  |  |  |  | X ^i^ |
| Abbreviations: CBC: Complete blood count; CSF, Cerebrospinal fluid; CT, Computed tomography; ctDNA, Circulating tumour DNA; DLT, dose-limiting toxicity; ECG, Electrocardiogram; ECOG, Eastern cooperative oncology group; IELSG, International Extranodal Lymphoma Study Group; KPS, Karnofsky performance status; LDH, Lactate dehydrogenase; MMSE, Mini-mental state examination; PET/CT, Positron Emission Tomography / Computed Tomography.  The window of visit in each course of study treatment is ± 7 days.  ^a^ Subjects will receive a total of 6 courses of treatment with sintilimab, rituximab, methotrexate, and temozolomide. After 4 courses of treatment, patients with CR, CRu or PR obtained in the mid-term evaluation continued to receive the original treatment, and those with PD were excluded from the group. After 6 courses of treatment, CR or CRu patients were evaluated at the end of the treatment period and entered the follow-up period. For patients with PR or PD, the investigator decided to rescue the treatment plan.  ^b^ Vital signs include systolic and diastolic blood pressure, heart rate, respiratory rate, pulse, blood oxygen saturation and body temperature.  ^c^ For premenopausal females only. Should be drawn within 2 weeks of initiating treatment.  ^d^ Within 2 week prior to initiation of treatment  ^e^ Scan should be done prior to next cycle (recommended within 14 days of last dose).  ^f^ DLT will be assessed through the first 3 weeks of the study.  ^g^ During follow-up, would be done if progression on imaging | | | | | | | | | | | | | | | | | | | | | | | | | | | |
| ^h^ Carry out lumbar puncture to retain cerebrospinal fluid when the patient's condition allows  ^i^ Within 2 years after the last subject was enrolled, for those who did not withdraw the informed consent form, they will collect the survival information of the subject (i.e., the date and reason of death, subsequent tumor treatment, etc.) by telephone and/or clinical visit after the final visit, once every 3 months in the first year, and once every 6 months in the second year, then annually for years 3 to 5, thereafter until the end of the study. | | | | | | | | | | | | | | | | | | | | | | | | | | | |

# 10.1 Safety Evaluation

The safety will be assessed by adverse events, laboratory test results, vital signs measurement, physical examination and ECOG performance status evaluation. Clinically relevant changes during the study must be recorded in the adverse events section of CRF. The investigator will track the clinically significant abnormalities that continue to exist at the end of the study/early withdrawal until the end point of clinical stability is resolved or reached. If there are clinical indications, safety monitoring and evaluation can be carried out more frequently. The incidence and severity of adverse events and serious adverse events during treatment will be counted and analyzed according to CTCAE V4.0 (Appendix 2).

# 10.2 Adverse Events and Serious Adverse Events

# 10.2.1 Adverse Events Definitions

An AE is any untoward medical occurrence in a study subject and does not necessarily have a causal relationship with this treatment.

An AE therefore can be any unfavorable and unintended sign (including laboratory finding), symptom or disease temporally associated with participation in an investigational study, whether or not considered drug-related. In addition to new events, any increase in the severity or frequency of a pre-existing condition that occurs after the subject signs a consent form for participation is considered an AE. This includes any side effect, injury, toxicity, or sensitivity reaction.

Whenever possible, the Common Terminology Criteria for Adverse Events (CTCAE) version 4.0 should be used to describe the event and for assessing the severity of AEs. Any events representing a change in the CTCAE Grade need to be recorded. This includes any change in laboratory values.

For AEs not adequately addressed in the CTCAE, the Table 2 may be used:

**Table 2. CTCAE Severity of Adverse Events**

| **Severity** | **Description** |
| --- | --- |
| GRADE 1 – Mild | Transient or mild discomfort; no limitation in activity; no medical intervention/therapy required. |
| GRADE 2 – Moderate | Mild to moderate limitation in activity—some assistance may be needed; no or minimal medical intervention/therapy required. |
| GRADE 3 – Severe | Marked limitation in activity, some assistance usually required; medical intervention/therapy required, hospitalizations possible. |
| GRADE 4 – Life-threatening | Extreme limitation in activity, significant assistance required; life-threatening (immediate risk of death); significant medical intervention/therapy required, hospitalization or hospice care probable. |
| GRADE 5 – Fatal | Death |

Any condition, laboratory abnormality, or physical finding with an onset date prior to the subject signing consent for study participation is considered to be pre-existing in nature and part of the subject’s medical history.

# 10.2.2 Serious Adverse Event

A serious adverse event is defined as adverse events that occur at any drug dose and meet any of the following criteria:

- Results in death
- Is life-threatening

(The subject was at risk of death at the time of the event. It does not refer to an event that hypothetically might have caused death if it were more severe.)

- Requires inpatient hospitalization or prolongation of existing hospitalization
- Results in persistent or significant disability/incapacity
- Is a congenital anomaly/birth defect
- Is a suspected transmission of any infectious agent via a medicinal product
- Is Medically Important*

*Medical and scientific judgment should be exercised in deciding whether expedited reporting is also appropriate in other situations, such as important medical events that may not be immediately life threatening or result in death or hospitalization but may jeopardize the subject or may require intervention to prevent one of the other outcomes listed in the definition above. These should usually be considered serious.

If a serious and unexpected adverse event occurs for which there is evidence suggesting a causal relationship between the study treatment and the event (eg, death from anaphylaxis), the event must be reported as a serious and unexpected suspected adverse reaction even if it is a component of the study endpoint (eg, all-cause mortality).

# 10.2.3 Causality

Using the following criteria, the relationship of the AE to the study drug should be assessed as follows:

- Yes: The event is suspected to be related if:

-There is a clinically plausible time sequence between onset of the AE and administration of study treatment; and/or

-There is a biologically plausible mechanism for the study treatment to cause or contribute to the AE; and/or

-The event responds to withdrawal of the study intervention (dechallenge) and/or recurs with rechallenge (when clinically feasible); and/or

-The AE cannot be reasonably attributed to concurrent/underlying illness,other drugs, or procedures

- No:

-The AE is more likely to be explained by the subject’s clinical state, underlying disease, concomitant medication, study or non-study procedure; and/or

-The time of occurrence of the AE is not reasonably related to administration of study treatment; and/or

-The event is unlikely to be related to the investigational procedures(s)

# 10.2.4 Adverse Events Reporting Procedures

All AEs (e.g., any new event or worsening in severity or frequency of a pre-existing condition or laboratory finding) with an onset date after the subject signs consent for study participation must be promptly documented on the appropriate summary. Details of the event must include severity, relationship to study drug, duration, action taken, and outcome.

All AEs that are considered related to study procedures must be followed to resolution or stabilization if improvement is not expected.

AEs should be reported from the time the subject signs consent through 30 days post-last study intervention. In addition, the investigator should report any AE that may occur after this time period that is believed to have a reasonable possibility of being associated with study intervention. If a subject discontinues study prior to receiving any study intervention, AEs must be reported through the end-of-study visit. AEs which completely resolve and then recur should be recorded as a new AE. For subjects who complete the end of study visit less than 30 days following the last study intervention, a follow up of ongoing AEs should be attempted by telephone, and documented in the subject’s source. AEs continuing at 30 days post-last treatment should have a comment in the source by the investigator that the event has stabilized or is not expected to improve.

The Principal Investigator is responsible for evaluating all AEs, obtaining supporting documents, and determining that documentation of the event is adequate. Adverse events will be assigned a severity grade using the NCI CTCAE grading scale v4.0 and recorded. Furthermore, the occurrence of grade 2 or higher immune-related adverse events will be collected and designated as immune-related events of clinical interest (irAEs).

# 10.3 Expected Side Effects and Supportive Care for Sintilimab and RMT

Refer to package insert for frequency of side effects of sintilimab, rituximab, methotrexate and temozolomide.

# 10.3.1 Dose Modification and Toxicity Management for Immune-related AEs Associated with Sintilimab

AEs associated with sintilimab exposure may represent an immunologic etiology. These immune-related AEs (irAEs) may occur shortly after the first dose or several months after the last dose of sintilimab treatment and may affect more than one body system simultaneously. Therefore, early recognition and initiation of treatment is critical to reduce complications. Based on existing clinical study data, most irAEs were reversible and could be managed with interruptions of sintilimab, administration of corticosteroids and/or other supportive care. For suspected irAEs, ensure adequate evaluation to confirm etiology or exclude other causes. Additional procedures or tests such as bronchoscopy, endoscopy, skin biopsy may be included as part of the evaluation. Based on the severity of irAEs, withhold or permanently discontinue sintilimab and administer corticosteroids. Dose modification and toxicity management guidelines for irAEs associated with sintilimab are provided in the table below (Table 2).

For any Grade 1 immune related adverse event (irAE), proceed with treatment of sintilimab and manage Grade 1 irAEs per investigator discretion unless otherwise stated in the table below (Table 3).

**Table 3. Dose modification and toxicity management guidelines for immune-related adverse events associated with sintilimab**

| **General instructions:** 1. Corticosteroid taper should be initiated upon AE improving to Grade 1 or less and continue to taper over at least 4 weeks. 2. For situations where sintilimab has been withheld, sintilimab can be resumed after AE has been reduced to Grade 1 or 0 and corticosteroid has been tapered.3. For severe and life-threatening irAEs, IV corticosteroid should be initiated first followed by oral steroid. Other immunosuppressive treatment should be initiated if irAEs cannot be controlled by corticosteroids. | | | | |
| --- | --- | --- | --- | --- |
|  |  |  |  |  |
|  |  |  |  |  |
|  |  |  |  |  |
|  |  |  |  |  |
|  |  |  |  |  |
|  |  |  |  |  |
| **Immune-related AEs** | **Toxicity grade or conditions**  **(CTCAE v4.0)** | **Action taken to sintilimab** | **irAE management with corticosteroid and/or other therapies** | **Monitor and follow-up** |
| Pneumonitis | Grade 2 | Withhold | - Administer corticosteroids (initial dose of 1-2 mg/kg prednisone or equivalent) followed by taper | - Monitor participants for signs and symptoms of pneumonitis - Evaluate participants with suspected pneumonitis with radiographic imaging and initiate corticosteroid treatment - Add prophylactic antibiotics for opportunistic infections |
|  | Grade 3 or 4, or recurrent Grade 2 | Permanently discontinue |  |  |
| Diarrhea / Colitis | Grade 2 or 3 | Withhold | - Administer corticosteroids (initial dose of 1-2 mg/kg prednisone or equivalent) followed by taper | - Monitor participants for signs and symptoms of enterocolitis (ie, diarrhea, abdominal pain, blood or mucus in stool with or without fever) and of bowel perforation (ie, peritoneal signs and ileus). - Participants with ≥ Grade 2 diarrhea suspecting colitis should consider GI consultation and performing endoscopy to rule out colitis. |
|  | Grade 4 | Permanently discontinue |  |  |
| AST / ALT elevation or Increased bilirubin | Grade 2 | Withhold | - Administer dose of corticosteroids (initial dose of 0.5-1 mg/kg prednisone or equivalent) followed by taper | - Monitor with liver function tests (consider weekly or more frequently) until liver enzyme value returned to baseline or is stable |
|  | Grade 3 or 4 | Withhold or permanently discontinue | - Administer dose of corticosteroids (initial dose of 1-2 mg/kg prednisone or equivalent) followed by taper |  |
| Type 1 diabetes mellitus (T1DM) or Hyperglycemia | Newly onset T1DM or Grade 3 or 4 hyperglycemia associated with evidence of β-cell failure | Withhold | - Initiate insulin replacement therapy for participants with T1DM - Administer anti-hyperglycemic in participants with hyperglycemia | - Monitor participants for hyperglycemia or other signs and symptoms of diabetes. |
| Hypophysitis | Grade 2 | Withhold | - Administer corticosteroids and initiate hormonal replacements as clinically indicated. | - Monitor for signs and symptoms of hypophysitis (including hypopituitarism and adrenal insufficiency) |
|  | Grade 3 or 4 | Withhold or permanently discontinue^1^ |  |  |
| Hyperthyroidism | Grade 2 | Continue | - Treat with non-selective beta- blockers (eg, propranolol) or thionamides as appropriate | - Monitor for signs and symptoms of thyroid disorders |
|  | Grade 3 or 4 | Withhold or permanently discontinue^1^ |  |  |
| Hypothyroidism | Grade 2-4 | Continue | - Initiate thyroid replacement hormones (eg, levothyroxine or liothyroinine) per standard of care | - Monitor for signs and symptoms of thyroid disorders. |
| Nephritis and Renal dysfunction | Grade 2 | Withhold | - Administer corticosteroids (prednisone 1-2 mg/kg or equivalent) followed by taper | - Monitor changes of renal function |
|  | Grade 3 or 4 | Permanently discontinue |  |  |
| Myocarditis | Grade 1 or 2 | Withhold | - Based on severity of AE administer corticosteroids | - Ensure adequate evaluation to confirm etiology and/or exclude other causes |
|  | Grade 3 or 4 | Permanently discontinue |  |  |
| All other immune- related AEs | Intolerable/ persistent Grade 2 | Withhold | - Based on type and severity of AE administer corticosteroids | - Ensure adequate evaluation to confirm etiology and/or exclude other causes |
|  | Grade 3 | Withhold or discontinue based on the type of event. Events that require discontinuation include and not limited to: Gullain-Barre Syndrome, encephalitis |  |  |
|  | Grade 4 or recurrent Grade 3 | Permanently discontinue |  |  |
| 1. Withhold or permanently discontinue sintilimab is at the discretion of the investigator or treating physician.   **NOTE:** For participants with Grade 3 or 4 immune-related endocrinopathy where withhold of sintilimab is required, sintilimab may be resumed when AE resolves to ≤ Grade 2 and is controlled with hormonal replacement therapy or achieved metabolic control (in case of T1DM). | | | | |

# 10.3.2 Dose Modification and Toxicity Management of Infusion-reactions Related to Sintilimab

Sintilimab may cause severe or life-threatening infusion-reactions including severe hypersensitivity or anaphylaxis. Signs and symptoms usually develop during or shortly after drug infusion and generally resolve completely within 24 hours of completion of infusion. Dose modification and toxicity management guidelines on sintilimab associated infusion reaction are provided in the Table 4.

**Table 4. Infusion Reaction Treatment Guidelines**

| **NCI CTCAE Grade** | **Treatment** | **Premedication at subsequent dosing** |
| --- | --- | --- |
| **Grade 1** Mild reaction; infusion interruption not indicated; intervention not indicated | Increase monitoring of vital signs as medically indicated until the subject is deemed medically stable in the opinion of the investigator. | None |
| **Grade 2** Requires infusion interruption but responds promptly to symptomatic treatment (e.g., antihistamines, NSAIDs, narcotics, IV fluids); prophylactic medications indicated for ≤24 hrs. | **Stop Infusion and monitor symptoms.** Additional appropriate medical therapy may include but is not limited to:  IV fluids  Antihistamines NSAIDs  Acetaminophen  Narcotics Increase monitoring of vital signs as medically indicated until the subject is deemed medically stable in the opinion of the investigator. If symptoms resolve within one hour of stopping drug infusion, the infusion may be restarted at 50% of the original infusion rate (e.g. from 100 mL/hr to 50 mL/hr). Otherwise dosing will be held until symptoms resolve and the subject should be premedicated for the next scheduled dose. **Subjects who develop Grade 2 toxicity despite adequate premedication should be permanently discontinued from further trial treatment administration.** | Subject may be premedicated 1.5h (± 30 minutes) prior to infusion of sintilimab with: Diphenhydramine 50 mg po (or equivalent dose of antihistamine). Acetaminophen 500- 1000 mg po (or equivalent dose of antipyretic). |
|
|
|
|
| **Grades 3 or 4** Grade 3: Prolonged (i.e., not rapidly responsive to symptomatic medication and/or brief interruption of infusion); recurrence of symptoms following initial improvement; hospitalization indicated for other clinical sequelae (e.g., renal impairment, pulmonary infiltrates) Grade 4: Life-threatening; pressor or ventilatory support indicated | **Stop Infusion.** Additional appropriate medical therapy may include but is not limited to:  IV fluids  Antihistamines  NSAIDs  Acetaminophen  Narcotics  Oxygen  Pressors  Corticosteroids  Epinephrine** Increase monitoring of vital signs as medically indicated until the subject is deemed medically stable in the opinion of the investigator. Hospitalization may be indicated. **In cases of anaphylaxis, epinephrine should be used immediately. **Subject is permanently discontinued from further trial treatment administration.** | No subsequent dosing |
|
|
|
|
|
|
|
|
|
| An appropriate resuscitation plan should in place and a physician readily available during the period of drug administration. For Further information, please refer to the Common Terminology Criteria for Adverse Events v4.0 (CTCAE) at https://evs.nci.nih.gov/ftp1/CTCAE/About.html. | | |
|

# 10.3.3 Dose Delay or Modification of Rituximab, HD-MTX and Temozolamide

| **Event(s)** | **Dose Delay or Modification** |
| --- | --- |
| Grade 3 or 4 neutropenia on Day 1 of any Cycle with or without infection or fever First delay | - Delay all study treatment. Treatment cannot be delayed for more than 2 weeks. - Administer growth factors as appropriate; (e.g., G-CSF for neutropenia as indicated and for all subsequent cycles). - If ANC recovers to ＞1000/μL by Day 7 of the scheduled date for the next cycle, administer full dose of sintilimab, rituximab, HD-MTX and temozolamide. - If ANC recovers to ＞1000/μL on or after Day 8 of the scheduled date for the next cycle, reduce the dose of HD-MTX to 1.0 g/m2. |
|
|
|
|
| Recurrent Grade 3 or 4 neutropenia on Day 1 of any Cycle | - Delay doses of all study treatment. Treatment cannot be delayed for more than 2 weeks. - If ANC recovers to ＞1000/μL by Day 7 of the scheduled date for the next cycle, administer full dose of study treatment. If ANC recovers to ＞1000/μL on or after Day 8 of the scheduled date for the next cycle, then:   - If the dose of HD-MTX is 1.0 g/m2, then reduce temozolamide to the next lowest dose level of 100 mg/m2 (1st dose reduction) and maintain HD-MTX dose of 1.0 g/m2. If there was a prior dose reduction, then reduce temozolamide to the next lowest dose level of 75 mg/m2 (2nd dose reduction) and maintain rituximab dose at 375 mg/m2.   - No dose reductions of rituximab for neutropenia are allowed. |
|
|
|
|
| Severe thrombocytopenia (platelets <20,000/μL) and/or symptomatic bleeding in patients who are not receiving concomitant anticoagulants or platelet inhibitors | - Delay the treatment in case of severe thrombocytopenia (platelets <20,000/μL) or symptomatic bleeding (irrespective of platelet count) until it resolves. - For patients who are on platelet inhibitors, when thrombocytopenia with platelets < 20,000/μL develops, consideration should be given to temporarily pause their use |
|
| Grade 3 or 4 thrombocytopenia on Day 1 of any cycle, first episode | - Delay doses of all study treatment. - If platelet count recovers to >75,000/μL by Day 7 of the scheduled date of the next cycle, administer full dose of study treatment. - If platelet count recovers to >75,000/μL on or after Day 8 of the scheduled date of the next cycle, reduce the dose of HD-MTX to the next (lowest) dose level (1.0 g/m2). |
|
|
| Recurrent Grade 3 or 4 thrombocytopenia | - Delay doses of all study treatment. - If platelet count recovers to >75,000/μL by Day 7 of the scheduled date of the next cycle, administer full dose of study treatment - If platelet count recovers to >75,000/μL on or after Day 8 of the scheduled date of the next cycle, then:   - If the dose of HD-MTX is 1.0 g/m2, then reduce temozolamide to the next lowest dose level of 100 mg/m2 (1st dose reduction) and maintain HD-MTX dose of 1.0 g/m2. If there was a prior dose reduction, then reduce temozolamide to the next lowest dose level of 75 mg/m2 (2nd dose reduction) and maintain HD-MTX dose at 1.0 g/m2.   - No more than 2 dose reductions of temozolamide are allowed.   - If patient develops Grade 4 thrombocytopenia following HD-MTX and temozolamide dose reductions, discontinue all study treatment permanently. |
|
|
|
|
|
| Grade 1 or 2 neutropenia and/or thrombocytopenia | No dose reduction or delay |
| Total Bilirubin > 3.0 mg/dL | - Delay treatment until resolution to ≤ 1.5 mg/dL within ≤ 14 days. Evaluate for causality. - Any case involving an increase in hepatic transaminase ＞ 3 × baseline AND an increase in direct bilirubin ＞ 2 × ULN, WITHOUT any findings of cholestasis or jaundice or signs of hepatic dysfunction AND in the absence of other contributory factors (e.g., concomitant exposure to known hepatotoxic agent or of a documented infectious etiology) is suggestive of potential drug-induced liver injury, and drug should be discontinued. |
| Grade 3 or 4 non-hematologic toxicity not specifically described above (excluding alopecia, nausea, and vomiting) | - Delay study treatment for a maximum of 14 days - If improvement to Grade ≤ 1 or baseline, continue study therapy at full dose, or dose reduce at the discretion of the investigator per site’s standard after discussion with the Medical Monitor. |
|
| Grade 2 non-hematologic toxicity | - Delay study treatment for a maximum of 14 days. - If improvement to Grade ≤ 1 or baseline, administer previous doses of study treatment. |
|
| Grade 1 non-hematologic toxicity | No dose reduction or delay |

# 10.3.4 Treatment Discontinuation Criteria

# 10.3.4.1 Sintilimab

A patient should permanently discontinue sintilimab if any of the following occur:

Grade 3 or 4 immune-relatedImmune-related AEs according to Table 3.

# 10.3.4.2 Rituximab

A patient should permanently discontinue rituximab if any of the following occur:

- Grade 4 infusion-related symptom or anaphylaxis. The patient should be withdrawn from study treatment immediately and supportive treatment given.
- Recurrence of Grade 3 infusion-related symptoms at re-challenge, regardless of timing (e.g., within same session or at the next session)
- If patient has Grade 3 wheezing, bronchospasm, or generalized urticaria at first occurrence.

# 10.3.4.3 HD-MTX and Temozolamide

A patient should permanently discontinue HD-MTX and temozolamide, if any of the following occur:

- Grade 3 or 4 hematologic toxicity that does not resolve to Grade 2 and delays treatment by 14 days despite administration of growth factors
- Grade 2 non-hematologic toxicity that does not resolve to Grade 1 or baseline value and delays treatment by 14 days
- Disease progression

# 10.3.5 Diet/Contraception/Other Considerations

# 10.3.5.1 Diet

Subjects should maintain normal diet, unless adjustment is needed to control adverse events, such as diarrhea, nausea or vomiting.

# 10.3.5.2 Contraception

Sintilimab, rituximab, high-dose methotrexate and temozolomide may have adverse effects on a fetus in utero. Furthermore, it is not known if sintilimab has transient adverse effects on the composition of sperm.

For this trial, male subjects will be considered to be of non-reproductive potential if they have azoospermia (whether due to having had a vasectomy or due to an underlying medical condition).

Female subjects will be considered of non-reproductive potential if they are either:

(1) postmenopausal (defined as at least 12 months with no menses without an alternative medical cause; in women < 45 years of age a high follicle stimulating hormone (FSH) level in the postmenopausal range may be used to confirm a post-menopausal state in women not using hormonal contraception or hormonal replacement therapy. In the absence of 12 months of amenorrhea, a single FSH measurement is insufficient.);

OR

(2) have had a hysterectomy and/or bilateral oophorectomy, bilateral salpingectomy or bilateral tubal ligation/occlusion, at least 6 weeks prior to screening;

OR

(3) has a congenital or acquired condition that prevents childbearing.

Female and male subjects of reproductive potential must agree to avoid becoming pregnant or impregnating a partner, respectively, while receiving study drug and for 120 days after the last dose of study drug by complying with one of the following:

(1)practice abstinence† from heterosexualactivity;

OR

1. use (or have their partner use) acceptable contraception during heterosexual activity.

Acceptable methods of contraception are:

Single method (one of the following is acceptable):

- intrauterine device (IUD)

 -vasectomy of a female subject’s male partner

 -contraceptive rod implanted into the skin

Combination method (requires use of two of the following):

-diaphragm with spermicide (cannot be used in conjunction with cervical cap/spermicide)

-cervical cap with spermicide (nulliparous women only)

-contraceptive sponge (nulliparous women only)

-male condom or female condom (cannot be used together)

-hormonal contraceptive: oral contraceptive pill (estrogen/progestin pill or progestin-only pill), contraceptive skin patch, vaginal contraceptive ring, or subcutaneous contraceptive injection

†Abstinence (relative to heterosexual activity) can be used as the sole method of contraception if it is consistently employed as the subject’s preferred and usual lifestyle and if considered acceptable by local regulatory agencies and ERCs/IRBs. Periodic abstinence (e.g., calendar, ovulation, sympto-thermal, post-ovulation methods, etc.) and withdrawal are not acceptable methods of contraception.

If a contraceptive method listed above is restricted by local regulations/guidelines, then it does not qualify as an acceptable method of contraception for subjects participating at sites in this country/region.

Subjects should be informed that taking the study medication may involve unknown risks to the fetus (unborn baby) if pregnancy were to occur during the study. In order to participate in the study subjects of childbearing potential must adhere to the contraception requirement (described above) from the day of study medication initiation (or 14 days prior to the initiation of study medication for oral contraception) throughout the study period up to 120 days after the last dose of trial therapy. If there is any question that a subject of childbearing potential will not reliably comply with the requirements for contraception, that subject should not be entered into the study.

# 10.3.5.3 Use in Pregnancy

All initial reports of pregnancy in female subjects or partners of male subjects must be reported to the sponsor by the study-site personnel within 24 hours of their knowledge of the event using the appropriate pregnancy notification form. Abnormal pregnancy outcomes (eg, spontaneous abortion, fetal death, stillbirth, congenital anomalies, and ectopic pregnancy) are considered serious adverse events and must be reported using the Serious Adverse Event Form. Any subject who becomes pregnant during the study must discontinue further study treatment. Follow-up information regarding the outcome of the pregnancy and any postnatal sequelae in the infant will be required.

# 10.3.5.4 Use in Nursing Women

It is unknown whether sintilimab is excreted in human milk. Since many drugs are excreted in human milk, and because of the potential for serious adverse reactions in the nursing infant, subjects who are breast-feeding are not eligible for enrollment.

# 10.4 Evaluation of Response

The efficacy evaluation was carried out according to the provisions of time and event schedule. The evaluation was conducted by Investigators according to the response criteria of the International PCNSL Collaborative Group. Response definition was based on changes in tumour size of enhanced lesions on gadolinium-enhanced MRI, ocular examination and CSF exams (Table 5). Baseline MRI was obtained in all patients before initiating therapy. In short, complete remission (CR) was defined as complete disappearance of all lesions, a partial response (PR) was defined as a ≥50% decrease in the size of the enhancing tumor, progressive disease (PD) was defined as a ≥25% increase in tumor size or the occurrence of a new lesion, and stable disease (SD)was defined as a situation that could not be classified as CR, PR, or PD. The imaging evaluation of all target lesions was evaluated by imaging experts. After completing treatment, the patients were followed up every 3 months in the first year, and then every 6 months for the second year, then annually for years 3 to 5. After progression, the survival status of patients was followed up every 3 months.

**Table 5. Response Criteria for Primary Central Nervous System Lymphoma**


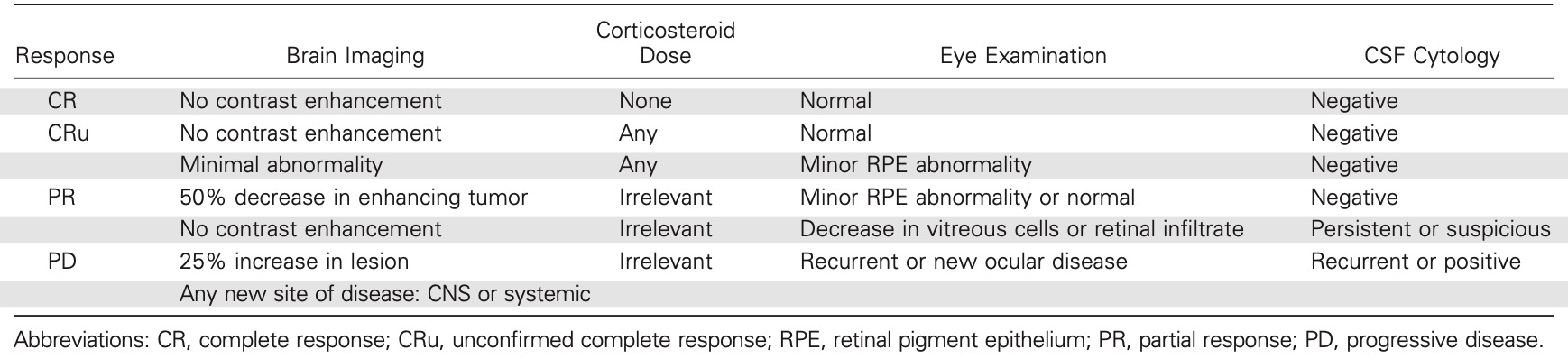


# 10.5 Evaluation of Biomarkers

The evaluation of biomarkers will focus on several objectives: 1) Evaluate the next generation sequencing (such as whole-exome sequencing) of biopsy tissue before treatment; 2) Evaluate the immunohistochemical detection of biopsy tissue before treatment (including but not limited to routine index detection: PD-1, PD-L1, tumor infiltrating lymphocyte related immunophenotype, etc.); 3) Evaluate tumor-specific cell-free DNA in cerebrospinal fluid and peripheral blood; 4) Evaluate the analysis of cytokines and related protein components in cerebrospinal fluid and peripheral blood; 5) Peripheral blood lymphocyte subpopulations, soluble PD-L1 level and immune group library; 6) FDG PET quantitative index; 7) Gadolinium-enhanced brain MRI quantitative index; and 8) Quality of life assessment, MMSE. Additional samples can be collected to help understand unexplained adverse events.

The analysis of biomarkers depends on the availability of appropriate biomarker determination. If it is obvious that the analysis will have no scientific value during or at the end of the study, or if there are not enough samples or enough responders to conduct appropriate biomarker evaluation, the analysis of biomarkers can be postponed or not carried out. If the study is terminated prematurely or the clinical efficacy is poor, the completion of biomarker evaluation will be based on the expected use of the data.

# 11 STATISTICAL METHODS

A comprehensive statistical analysis plan will be prepared prior to first subject enrolled. All variables will use appropriate descriptive statistics. Continuous variables will be summarized by mean, median and standard error. Classification variables will be summarized by frequency count and percentage of each category. Continuous variables will be summarized using the number of observations, mean, standard deviation, coefficient of variation, median, and range as appropriate. Categorical values will be summarized using the number of observations and percentages as appropriate. The efficacy and safety data will be summarized from the study.

# 11.1 Sample Size Determination

This study is a single-arm trial. The proportion of patients who have achieved remission after treatment is the primary objective (overall response rate, ORR). In Part A of the study, the first 6 patients will be treated with sintilimab, rituximab, HD-MTX and temozolomide in the safety run-in phase, and will be closely followed up and evaluated for DLTs. If 1 or fewer patients experience dose-limiting toxicity (DLT), enrollment will continue according to the Simon’s two-stage design described below. If >1 DLTs are observed, dose modifications will be considered before moving forward.

According to previous study [36], the ORR of RMT treatment for newly PCNSL was about 58%, and it is estimated that the ORR of sintilimab plus RMT treatment in this study is 83%. Although the true overall response rate to sintilimab plus RMT may be less than 83%, it is expected that this protocol adds PD-1 antibody but reduces the MTX dose, which is more convenient and more tolerant than the previous regimen, so we will accept any protocol higher than 58%. To this end, Simon’s Optimal two-stage design will utilize a null (non-promising) response rate of 58% and an alternative (promising) rate of 83%.

In the first stage, we will enroll 8 eligible patients. If 5 or fewer patients achieve PR, no additional patients will be enrolled and the regimen will be considered not promising. If >5 patients achieve PR, then an additional 19 patients will be enrolled for the second stage. If more than 19 out of a total of 27 patients achieve PR, this treatment regimen will be declared promising. This decision rule has a one-side type I error (declaring the new treatment regimen promising while it is actually not) rate of 0.05 and a type II error (declaring the new treatment regimen not promising while it actually is) rate of 0.20. The first 6 patients enrolled onto the safety portion of the study will be included in the analysis for efficacy.

Part B of this study will enroll additional patients for expansion. The primary outcome for the expansion cohort is PFS. Based on previous study of PCNSL patients receiving two drug combination (HD-MTX and temozolomide) alone but without consolidation treatment suggesting 2-year PFS of roughly 25% [44], and in the absence of adequate historical control, the sample size was calculated to show an improvement of 25% in the 2-year PFS for sintilimab plus RMT regimen. A one-arm trial that included 20 patients is required to reject the null hypothesis of a 2-year PFS of 25% under the alternative hypothesis that the true 2-year PFS is 50%, with a two-sided 5% significance level and 80% power.

Considering dropouts and withdrawals, the total sample size of this study is about 50 subjects.

# 11.2 Efficacy Analyses

Overall response rate (ORR) is defined as the proportion of subjects who achieve a PR or better according to the IPCG response criteria.

Complete response rate (CR) is defined as the proportion of subjects who achieve a CR or CRu according to the IPCG response criteria.

Duration of response (DOR) will be calculated among responders (with a PR or better response) from the date of initial documentation of a response (PR or better) to the date of first documented evidence of progressive disease, as defined in the IPCG response criteria.

Progression-free survival (PFS) defined as the time from the date of the initial treatment to the date of first documented disease progression, as defined in the IPCG response criteria, or death due to any cause, whichever occurs first.

Overall survival (OS) is measured from the date of the initial treatment to the date of the subject’s death. If the subject is alive or the vital status is unknown, then the subject’s data will be censored at the date the subject was last known to be alive.

The distribution (median and Kaplan-Meier curves) of DOR will be provided using Kaplan- Meier estimates for subjects who achieved response during the study. Similar analysis will be performed for PFS and OS.

# 11.3 Safety Analyses

The baseline value for safety assessment is defined as the value collected at the time closest to, but prior to, the start of sintilimab plus RMT treatment. In order to evaluate the safety, the first six patients will be treated and their dose limiting toxicity (DLT) will be observed. The safety parameters to be evaluated are the incidence, severity, and type of adverse events, clinically significant changes in the subject’s physical examination findings, vital signs measurements, and clinical laboratory results. Exposure to investigational product and reasons for discontinuation of study treatment will be tabulated. Adverse events will be summarized by system organ class, preferred term, worst grade experienced by the subject, and by dose level.

# 12 SUPPORTING DOCUMENTS AND PRECAUTIONS

# 12.1 Informed Consent Process

Informed consent should be completed before the subjects agreed to participate in the study and continue throughout the study process. The informed consent is approved by the ethics committee, and the research object should read the informed consent form. The investigator will explain the research process and answer the questions raised by the subjects, and inform the subjects of the possible risks and their rights. Subjects can discuss with their families or guardians before agreeing to participate. The investigator must inform the subjects that it is voluntary to participate in the study and can withdraw from the study at any time. A copy of the informed consent form can be provided to the subject for storage. The rights and welfare of the subjects will be protected, and the quality of their medical care will not be affected by their refusal to participate in the study.

# 12.2 Privacy of Personal Data

The collection and processing of personal data from subjects enrolled in this study will be limited to those data that are necessary to fulfill the objectives of the study.

These data must be collected and processed with adequate precautions to ensure confidentiality and compliance with applicable data privacy protection laws and regulations. Appropriate technical and organizational measures to protect the personal data against unauthorized disclosures or access, accidental or unlawful destruction, or accidental loss or alteration must be put in place.

The informed consent obtained from the subject includes explicit consent for the processing of personal data and for the investigator/institution to allow direct access to his or her original medical records (source data/documents) for study-related monitoring, audit, and regulatory inspection. Investigators have the obligation to protect the data of research objects, and shall not disclose any subject information to unauthorized third parties without approval.

# 12.3 Collection and Use of Specimens and Data

Collect and use samples and data according to GCP requirements.

# 12.4 Quality Control and Quality Assurance

Conduct quality control and quality assurance according to GCP requirements.

# 12.5 Data Processing and Record Storage

# 12.5.1 Data Collection and Management

Data collection is conducted by clinical researchers under the supervision of the person in charge, who will be responsible for the accuracy, completeness and timeliness of the reported data. All data shall be clear to ensure accurate interpretation and traceability. The clinical data will be stored in a database, which should be protected by password, and a logical proofreading procedure should be set up when the database is established.

# 12.5.2 Research Data Retention

All data and original documents of the study shall be retained for at least 5 years after the end of the study.

# 12.6 Statement of Conflict of Interest

None.

# 13 REFERENCES

1. Grommes C, DeAngelis LM. Primary CNS Lymphoma. J Clin Oncol. 2017;35:2410–8.

2. Schabet M. Epidemiology of primary CNS lymphoma. J Neurooncol. 1999;43:199–201.

3. Panageas KS, Elkin EB, DeAngelis LM, Ben-Porat L, Abrey LE. Trends in survival from primary central nervous system lymphoma, 1975-1999: a population-based analysis. Cancer. 2005;104:2466–72.

4. Coté TR, Manns A, Hardy CR, Yellin FJ, Hartge P. Epidemiology of brain lymphoma among people with or without acquired immunodeficiency syndrome. AIDS/Cancer Study Group. J Natl Cancer Inst. 1996;88:675–9.

5. Gopal S, Patel MR, Yanik EL, Cole SR, Achenbach CJ, Napravnik S, et al. Temporal trends in presentation and survival for HIV-associated lymphoma in the antiretroviral therapy era. J Natl Cancer Inst. 2013;105:1221–9.

6. O’Neill BP, Decker PA, Tieu C, Cerhan JR. The changing incidence of primary central nervous system lymphoma is driven primarily by the changing incidence in young and middle-aged men and differs from time trends in systemic diffuse large B-cell non-Hodgkin’s lymphoma. Am J Hematol. 2013;88:997–1000.

7. Fallah J, Qunaj L, Olszewski AJ. Therapy and outcomes of primary central nervous system lymphoma in the United States: analysis of the National Cancer Database. Blood Adv. 2016;1:112–21.

8. Lauw MIS, Lucas C-HG, Ohgami RS, Wen KW. Primary Central Nervous System Lymphomas: A Diagnostic Overview of Key Histomorphologic, Immunophenotypic, and Genetic Features. Diagnostics (Basel). 2020;10:E1076.

9. Montesinos-Rongen M, Brunn A, Bentink S, Basso K, Lim WK, Klapper W, et al. Gene expression profiling suggests primary central nervous system lymphomas to be derived from a late germinal center B cell. Leukemia. 2008;22:400–5.

10. Lim DH, Kim WS, Kim SJ, Yoo HY, Ko YH. Microarray Gene-expression Profiling Analysis Comparing PCNSL and Non-CNS Diffuse Large B-Cell Lymphoma. Anticancer Res. 2015;35:3333–40.

11. Braggio E, Van Wier S, Ojha J, McPhail E, Asmann YW, Egan J, et al. Genome-Wide Analysis Uncovers Novel Recurrent Alterations in Primary Central Nervous System Lymphomas. Clin Cancer Res. 2015;21:3986–94.

12. Chapuy B, Roemer MGM, Stewart C, Tan Y, Abo RP, Zhang L, et al. Targetable genetic features of primary testicular and primary central nervous system lymphomas. Blood. 2016;127:869–81.

13. Langner-Lemercier S, Houillier C, Soussain C, Ghesquières H, Chinot O, Taillandier L, et al. Primary CNS lymphoma at first relapse/progression: characteristics, management, and outcome of 256 patients from the French LOC network. Neuro Oncol. 2016;18:1297–303.

14. Nelson DF, Martz KL, Bonner H, Nelson JS, Newall J, Kerman HD, et al. Non-Hodgkin’s lymphoma of the brain: can high dose, large volume radiation therapy improve survival? Report on a prospective trial by the Radiation Therapy Oncology Group (RTOG): RTOG 8315. Int J Radiat Oncol Biol Phys. 1992;23:9–17.

15. Shibamoto Y, Ogino H, Hasegawa M, Suzuki K, Nishio M, Fujii T, et al. Results of radiation monotherapy for primary central nervous system lymphoma in the 1990s. Int J Radiat Oncol Biol Phys. 2005;62:809–13.

16. Nabors LB, Portnow J, Ammirati M, Baehring J, Brem H, Brown P, et al. Central Nervous System Cancers, Version 1.2015. J Natl Compr Canc Netw. 2015;13:1191–202.

17. Batchelor T, Carson K, O’Neill A, Grossman SA, Alavi J, New P, et al. Treatment of primary CNS lymphoma with methotrexate and deferred radiotherapy: a report of NABTT 96-07. J Clin Oncol. 2003;21:1044–9.

18. Herrlinger U, Küker W, Uhl M, Blaicher H-P, Karnath H-O, Kanz L, et al. NOA-03 trial of high-dose methotrexate in primary central nervous system lymphoma: final report. Ann Neurol. 2005;57:843–7.

19. Ferreri AJM, Reni M, Foppoli M, Martelli M, Pangalis GA, Frezzato M, et al. High-dose cytarabine plus high-dose methotrexate versus high-dose methotrexate alone in patients with primary CNS lymphoma: a randomised phase 2 trial. Lancet. 2009;374:1512–20.

20. Wang X, Huang H, Bai B, Cai Q, Cai Q, Gao Y, et al. Clinical outcomes of patients with newly diagnosed primary central nervous system lymphoma are comparable on treatment with high-dose methotrexate plus temozolomide and with high-dose methotrexate plus cytarabine: a single-institution experience. Leuk Lymphoma. 2014;55:2497–501.

21. Rubenstein JL, Hsi ED, Johnson JL, Jung S-H, Nakashima MO, Grant B, et al. Intensive chemotherapy and immunotherapy in patients with newly diagnosed primary CNS lymphoma: CALGB 50202 (Alliance 50202). J Clin Oncol. 2013;31:3061–8.

22. Ferreri AJM, Cwynarski K, Pulczynski E, Ponzoni M, Deckert M, Politi LS, et al. Chemoimmunotherapy with methotrexate, cytarabine, thiotepa, and rituximab (MATRix regimen) in patients with primary CNS lymphoma: results of the first randomisation of the International Extranodal Lymphoma Study Group-32 (IELSG32) phase 2 trial. Lancet Haematol. 2016;3:e217-227.

23. Jahnke K, Korfel A, Martus P, Weller M, Herrlinger U, Schmittel A, et al. High-dose methotrexate toxicity in elderly patients with primary central nervous system lymphoma. Ann Oncol. 2005;16:445–9.

24. Omuro AMP, Taillandier L, Chinot O, Carnin C, Barrie M, Hoang-Xuan K. Temozolomide and methotrexate for primary central nervous system lymphoma in the elderly. J Neurooncol. 2007;85:207–11.

25. Batchelor TT, Grossman SA, Mikkelsen T, Ye X, Desideri S, Lesser GJ. Rituximab monotherapy for patients with recurrent primary CNS lymphoma. Neurology. 2011;76:929–30.

26. Maza S, Kiewe P, Munz DL, Korfel A, Hamm B, Jahnke K, et al. First report on a prospective trial with yttrium-90-labeled ibritumomab tiuxetan (Zevalin) in primary CNS lymphoma. Neuro Oncol. 2009;11:423–9.

27. Holdhoff M, Ambady P, Abdelaziz A, Sarai G, Bonekamp D, Blakeley J, et al. High-dose methotrexate with or without rituximab in newly diagnosed primary CNS lymphoma. Neurology. 2014;83:235–9.

28. Bromberg JEC, Issa S, Bakunina K, Minnema MC, Seute T, Durian M, et al. Rituximab in patients with primary CNS lymphoma (HOVON 105/ALLG NHL 24): a randomised, open-label, phase 3 intergroup study. Lancet Oncol. 2019;20:216–28.

29. Jiang X, Wang J, Deng X, Xiong F, Ge J, Xiang B, et al. Role of the tumor microenvironment in PD-L1/PD-1-mediated tumor immune escape. Mol Cancer. 2019;18:10.

30. Four M, Cacheux V, Tempier A, Platero D, Fabbro M, Marin G, et al. PD1 and PDL1 expression in primary central nervous system diffuse large B-cell lymphoma are frequent and expression of PD1 predicts poor survival. Hematol Oncol. 2017;35:487–96.

31. Cho H, Kim SH, Kim S-J, Chang JH, Yang W-I, Suh C-O, et al. Programmed cell death 1 expression is associated with inferior survival in patients with primary central nervous system lymphoma. Oncotarget. 2017;8:87317–28.

32. Qiu Y, Li Z, Pouzoulet F, Vishnu P, Copland III JA, Knutson KL, et al. Immune checkpoint inhibition by anti-PDCD1 (anti-PD1) monoclonal antibody has significant therapeutic activity against central nervous system lymphoma in an immunocompetent preclinical model. British Journal of Haematology. 2018;183:674–8.

33. Nayak L, Iwamoto FM, LaCasce A, Mukundan S, Roemer MGM, Chapuy B, et al. PD-1 blockade with nivolumab in relapsed/refractory primary central nervous system and testicular lymphoma. Blood. 2017;129:3071–3.

34. Terziev D, Hutter B, Klink B, Stenzinger A, Stögbauer F, Glimm H, et al. Nivolumab maintenance after salvage autologous stem cell transplantation results in long-term remission in multiple relapsed primary CNS lymphoma. Eur J Haematol. 2018;101:115–8.

35. Hoy SM. Sintilimab: First Global Approval. Drugs. 2019;79:341–6.

36. Wieduwilt MJ, Valles F, Issa S, Behler CM, Hwang J, McDermott M, et al. Immunochemotherapy with intensive consolidation for primary CNS lymphoma: a pilot study and prognostic assessment by diffusion-weighted MRI. Clin Cancer Res. 2012;18:1146–55.

37. Glass J, Won M, Schultz CJ, Brat D, Bartlett NL, Suh JH, et al. Phase I and II Study of Induction Chemotherapy With Methotrexate, Rituximab, and Temozolomide, Followed By Whole-Brain Radiotherapy and Postirradiation Temozolomide for Primary CNS Lymphoma: NRG Oncology RTOG 0227. J Clin Oncol. 2016;34:1620–5.

38. Hoang-Xuan K, Taillandier L, Chinot O, Soubeyran P, Bogdhan U, Hildebrand J, et al. Chemotherapy alone as initial treatment for primary CNS lymphoma in patients older than 60 years: a multicenter phase II study (26952) of the European Organization for Research and Treatment of Cancer Brain Tumor Group. J Clin Oncol. 2003;21:2726–31.

39. Wang S, Yao F, Lu X, Li Q, Su Z, Lee J-H, et al. Temozolomide promotes immune escape of GBM cells via upregulating PD-L1. Am J Cancer Res. 2019;9:1161–71.

40. Dai B, Qi N, Li J, Zhang G. Temozolomide combined with PD-1 Antibody therapy for mouse orthotopic glioma model. Biochem Biophys Res Commun. 2018;501:871–6.

41. Damsky W, Jilaveanu L, Turner N, Perry C, Zito C, Tomayko M, et al. B cell depletion or absence does not impede anti-tumor activity of PD-1 inhibitors. J Immunother Cancer. 2019;7:153.

42. Zinzani PL, Ribrag V, Moskowitz CH, Michot J-M, Kuruvilla J, Balakumaran A, et al. Safety and tolerability of pembrolizumab in patients with relapsed/refractory primary mediastinal large B-cell lymphoma. Blood. 2017;130:267–70.

43. Westin JR, Chu F, Zhang M, Fayad LE, Kwak LW, Fowler N, et al. Safety and Activity of PD1 Blockade by Pidilizumab in Combination with Rituximab in Patients with Relapsed Follicular Lymphoma: a Single Group, Open-label, Phase 2 Trial. Lancet Oncol. 2014;15:69–77.

44. Omuro A, Chinot O, Taillandier L, Ghesquieres H, Soussain C, Delwail V, et al. Methotrexate and temozolomide versus methotrexate, procarbazine, vincristine, and cytarabine for primary CNS lymphoma in an elderly population: an intergroup ANOCEF-GOELAMS randomised phase 2 trial. Lancet Haematol. 2015;2:e251-259.

# 14 APPENDICES

**Appendix 1: ECOG Performance Status**

| GRADE | ECOG |
| --- | --- |
| 0  1  2  3  4  5 | Fully active, able to carry on all pre-disease performance without restriction. Restricted in physically strenuous activity but ambulatory and able to carry out work of a light or sedentary nature, e.g., light house work, office work. Ambulatory and capable of all self-care but unable to carry out any work activities. Up and about more than 50% of waking hours. Capable of only limited self-care, confined to bed or chair more than 50% of waking hours. Completely disabled. Cannot carry on any self-care. Totally confined to bed or chair. Dead. |

**Appendix 2: National Cancer Institute Common Terminology Criteria for Adverse Events**

Common Terminology Criteria for Adverse Events (CTCAE) of the National Cancer Institute (NCI), Version 4.03

https://evs.nci.nih.gov/ftp1/CTCAE/About.html
